# A novel nuclear receptor subfamily enlightens the origin of heterodimerization

**DOI:** 10.1186/s12915-022-01413-0

**Published:** 2022-10-05

**Authors:** Brice Beinsteiner, Gabriel V. Markov, Maxime Bourguet, Alastair G. McEwen, Stéphane Erb, Abdul Kareem Mohideen Patel, Fatima Z. El Khaloufi El Khaddar, Claire Lecroisey, Guillaume Holzer, Karim Essabri, Isabelle Hazemann, Ali Hamiche, Sarah Cianférani, Dino Moras, Vincent Laudet, Isabelle M. L. Billas

**Affiliations:** 1 IGBMC (Institute of Genetics and of Molecular and Cellular Biology), Illkirch, France; 2 Université de Strasbourg, Illkirch, France; 3 Institut National de la Santé et de la Recherche Médicale (INSERM) U1258, Illkirch, France; 4 Centre National de la Recherche Scientifique (CNRS) UMR 7104, Illkirch, France; 5 Sorbonne Université, CNRS, UMR 8227, Integrative Biology of Marine Models, (LBI2M, UMR8227), Station Biologique de Roscoff (SBR), 29680 Roscoff, France; 6 Laboratoire de Spectrométrie de Masse BioOrganique, Université de Strasbourg, CNRS, IPHC UMR 7178, 67000 Strasbourg, France; 7 Infrastructure Nationale de Protéomique ProFI – FR2048 CNRS CEA, 67087 Strasbourg, France; 8 Ecole Normale Supérieure de Lyon, Université de Lyon, Institut de Génomique Fonctionnelle de Lyon, UMR 5242 CNRS, Molecular Zoology Team, 46 allée d’Italie, 69364 Lyon, Cedex 07 France; 9 Present address: Uniklinikum RWTH Aachen, Pauwelsstraße 30, 52074 Aachen, Nordrhein-Westfalen Germany; 10 Marine Eco-Evo-Devo Unit, Okinawa Institute of Science and Technology, 1919-1 Tancha, Onna-son, Okinawa, 904-0495 Japan; 11 Marine Research Station, Institute of Cellular and Organismic Biology, Academia Sinica, 23-10, Dah-Uen Rd, Jiau Shi, I-Lan, 262 Taiwan

**Keywords:** Amphioxus, Non-model animals, Nuclear receptor phylogeny, Nuclear receptor dimerization, NR7; Crystal structure; Native mass spectrometry

## Abstract

**Background:**

Nuclear receptors are transcription factors of central importance in human biology and associated diseases. Much of the knowledge related to their major functions, such as ligand and DNA binding or dimerization, derives from functional studies undertaken in classical model animals. It has become evident, however, that a deeper understanding of these molecular functions requires uncovering how these characteristics originated and diversified during evolution, by looking at more species. In particular, the comprehension of how dimerization evolved from ancestral homodimers to a more sophisticated state of heterodimers has been missing, due to a too narrow phylogenetic sampling. Here, we experimentally and phylogenetically define the evolutionary trajectory of nuclear receptor dimerization by analyzing a novel NR7 subgroup, present in various metazoan groups, including cnidarians, annelids, mollusks, sea urchins, and amphioxus, but lost in vertebrates, arthropods, and nematodes.

**Results:**

We focused on NR7 of the cephalochordate amphioxus *B. lanceolatum*. We present a complementary set of functional, structural, and evolutionary analyses that establish that NR7 lies at a pivotal point in the evolutionary trajectory from homodimerizing to heterodimerizing nuclear receptors. The crystal structure of the NR7 ligand-binding domain suggests that the isolated domain is not capable of dimerizing with the ubiquitous dimerization partner RXR. In contrast, the full-length NR7 dimerizes with RXR in a DNA-dependent manner and acts as a constitutively active receptor. The phylogenetic and sequence analyses position NR7 at a pivotal point, just between the basal class I nuclear receptors that form monomers or homodimers on DNA and the derived class II nuclear receptors that exhibit the classical DNA-independent RXR heterodimers.

**Conclusions:**

Our data suggest that NR7 represents the “missing link” in the transition between class I and class II nuclear receptors and that the DNA independency of heterodimer formation is a feature that was acquired during evolution. Our studies define a novel paradigm of nuclear receptor dimerization that evolved from DNA-dependent to DNA-independent requirements. This new concept emphasizes the importance of DNA in the dimerization of nuclear receptors, such as the glucocorticoid receptor and other members of this pharmacologically important oxosteroid receptor subfamily. Our studies further underline the importance of studying emerging model organisms for supporting cutting-edge research.

**Supplementary Information:**

The online version contains supplementary material available at 10.1186/s12915-022-01413-0.

## Background

Nuclear receptors (NRs) form a superfamily of DNA-binding transcription factors involved in cell growth and differentiation, embryonic development, and metabolism [1, 2]. The specificity of NRs lies in the activation of their transcriptional activity by small lipophilic ligands, which provide a direct link between the cellular environment and gene regulation [1]. A striking feature of the NR superfamily is their combinatorial mode of action that allows them to blend specificity and plasticity in the repertoire of genes they regulate. This provides a great flexibility in the transcriptional regulation by NRs, hence making them major physiological regulators ideal to fine tune energy expenditure and metabolism to the constantly changing needs of complex multicellular organisms. How such a delicate system has arisen during evolution, however, remains largely unknown.

All NRs share a common modular structure composed of a highly conserved DNA-binding domain (DBD) and a less conserved ligand-binding domain (LBD). These two domains are connected by a flexible hinge that plays an important role in the selection of DNA binding sites [2]—regulatory regions of target genes onto which NRs bind as homodimers, heterodimers or, more rarely, as monomers [3, 4]. NRs have been classified into two functional classes (class I and class II NRs) based on the conservation of amino acid residues in their LBD that connect the dimerization region with the ligand-binding pocket and the coregulator binding site [1]. Importantly, these two functional classes correlate with the two main functional NR types, namely class I NRs that form homodimers and comprise receptors such as the Retinoid X Receptors (RXRs) or the steroid hormone receptors (GR, PR, ERs), and class II NRs that function as heterodimers with RXR and include NRs such as the thyroid hormone receptors (TRs) and the retinoic acid receptors (RARs).

NRs form an ancient and conserved gene family that arose early in metazoans [5–7]. Phylogenetic analyses have proposed that one or two NRs were present at the base of metazoans (which is still true in most sponges), 25 at the base of bilaterians and 23 at the base of chordates [6, 7]. Based on these analyses, the superfamily has classically been divided into six distinct subfamilies (numbered NR1 through NR6) and an additional heterogenous “subfamily” (called NR0), which contains all receptors lacking typical NR domains [3, 4, 8]. The analysis of complete genome sequences available in a number of animal species, including early metazoans such as sponges, placozoans, or cnidarians has allowed the unveiling of the first steps of NR diversification [7]. As a consequence, the root of the NR tree could be positioned within subfamily II which in particular contains RXR, HNF4, and the COUP Transcription Factor 1 (COUP-TF) and that therefore is not monophyletic. This view separates the family into HNF4 on the one hand and all the other NRs on the other hand.

Major events in the molecular evolution of this gene family, including the timing of gene duplications and losses, have already been characterized [6, 7]. Nevertheless, some important questions concerning the earliest steps of NR diversification still remain open [9], in particular the way by which the major functions of NR proteins, such as ligand binding, DNA binding or dimerization, originated and diversified. For ligand binding, an evolutionary scenario was proposed, where the ancestral NR is suggested to be a sensor molecule capable of binding fatty acids with low affinity and low selectivity [7, 10, 11]. However, a similar evolutionary analysis has not yet been carried out to unveil the origin of the dimerization properties. Recently, we uncovered a previously unrecognized structural motif, the π-turn, whose evolution parallels the evolutionary diversification of nuclear receptor dimerization abilities from ancestral homodimers to derived heterodimers [12]. This motif is considered as a structural exaptation that enlarged the functional repertoire of NRs and therefore promoted their later diversification. However, how the range of dimerization abilities actually increased in relation to other basic functions such as DNA binding remained poorly understood.

Due to the growing number of available NR sequences, the NR phylogeny is constantly being refined and the latest studies with extensive species sampling revealed the existence of a new subgroup, initially called “invertebrate NRs” [7, 9, 13]. This group of sequences, located at the base of the highly diverse NR1 and NR4 subfamilies remained to be further analyzed as this might provide novel insights into the diversification of functional properties of NRs, in particular dimerization.

By analyzing these unclassified NR sequences from emerging model organisms, we could define a new subfamily that we named NR7 [9]. We characterized members of this new subfamily from various bilaterians and cnidarians and show that NR7 sequences were lost in the genomes of vertebrates, arthropods, and nematodes. Our data indicate that NR7 receptors are the remnant of an ancient NR subfamily, hence offering a unique window into the first steps of NR diversification. Through a detailed structural, functional, and evolutionary analysis of the NR7 from the cephalochordate amphioxus (*Branchiostoma lanceolatum*), we established that NR7 is a constitutive transcriptional activator that dimerizes with RXR in a DNA-dependent manner. This mechanism of action differs strikingly from more classical NRs that rely on a strong LBD dimerization interface and are mainly independent of the presence of DNA [2]. From our structural and phylogenetic analyses, we can infer that amphioxus NR7 represents the closest representative of the ancestral receptor that was first capable of heterodimerization with RXR, the ubiquitous partner of all NRs. As such, NR7 encompasses the main features of the more ancestral class I NRs, but also exhibits some of the signatures of class II NRs that were acquired in a stepwise manner during evolution towards full class II NRs. Thus, NR7 represents an important piece of the puzzle for the understanding of the evolution of NR heterodimerization. We propose that DNA-dependent heterodimerization was an intermediary step instrumental for the later establishment of the full combinatorial ability of NRs through RXR DNA-independent heterodimerization.

## Results

### NR7 defines an ancient NR subfamily located at a pivotal location in NR phylogeny

**Fig. 1:**
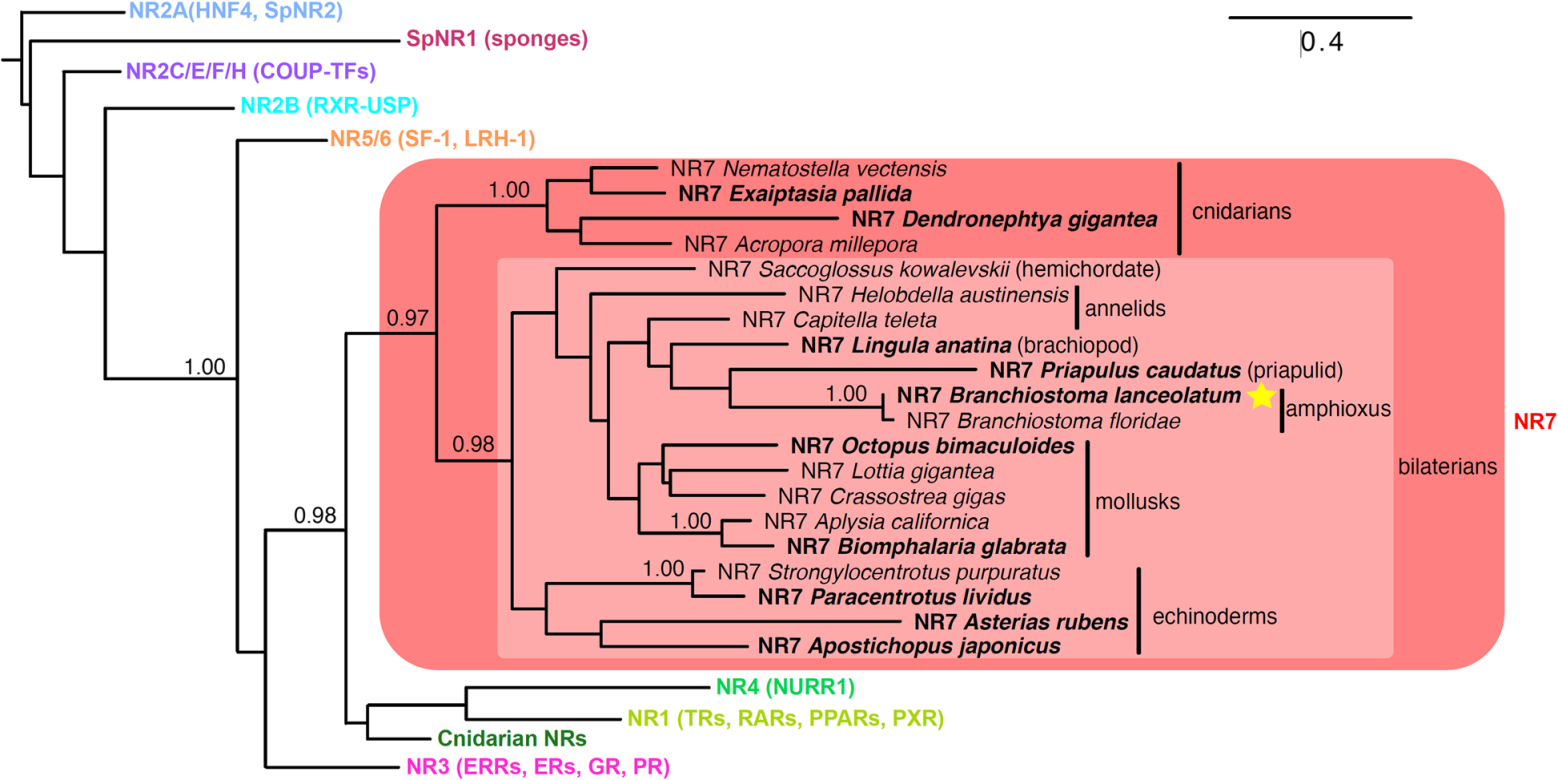
Phylogenetic position of the NR7 subfamily. A maximum likelihood tree of nuclear receptors (NRs). Classical NR subfamilies are simplified for clarity, and the full topology is indicated only for the bilaterian NR7 subfamily and their close cnidarian relatives. Branch support values are assessed by approximate likelihood-ratio test (aLRT) and are shown only if superior to 0.97, which is considered fully robust. Sequences in bold are new compared to 7. The amphioxus sequence characterized in this paper is indicated by a yellow star. The full tree is available in Additional file 1: Fig. S2, and accession numbers are provided in Additional file 1: Table S1

In order to delineate the position of the known NR orthologues within the NR superfamily, we sought of assessing their phylogenetic distribution. Using protein BLAST searches, we identified 10 additional members of the NR7 family (in bold in Fig. 1), including members of two phyla, where they were not previously reported, brachiopods and priapulids. In a phylogenetic tree, these sequences robustly grouped together with an approximate likelihood-ratio test (aLRT) value of 0.98 (Fig. 1, Additional file 1: Fig. S1, Additional file 1: Fig. S2 and Additional file 1: Suppl. Data S1). In addition, we found 4 NR7 sequences in cnidarians, grouping together with an aLRT value of 1.00. Our extended analysis shows that orthologs of NR7 are totally absent in vertebrates, arthropods, or nematodes, strongly suggesting that this gene was independently lost in these lineages which contain the main animal model organisms. This may explain why this new subfamily has escaped attention until now.

In agreement with this wide phylogenetical distribution and ancient conservation, we observed using in situ hybridization that amphioxus NR7 exhibits a complex expression pattern during development indicative of an important developmental function (Additional file 1: Fig. S3). We observed a ubiquitous maternal expression maintained at blastula-stage embryos followed by a zygotic expression in the anterior ectoderm at gastrula stages. In neurula-stage embryos, AmphiNR7 is expressed in the endoderm before becoming restricted to specific pharyngeal structures, such as the club-shaped gland and the pre-oral pit, at larval stages. Starting in late neurula, the gene is further expressed in the cerebral vesicle in the anterior central nervous system.

The phylogenetic position of NR7 within the superfamily is really remarkable. Indeed, NR7 is a robust (aLRT value of 0.97) sister group of the NR1 + NR4 subfamilies that contain the class II receptors that heterodimerize with RXR, plus some still uncharacterized sequences from cnidarians. Together, the ensemble grouping NR1+NR4 and NR7 is related to the NR3 subfamily and then, to a lesser extent, to NR5/NR6 (aLRT value of 0.99). From this placement, it appears that NR7 could be the first representative of NRs that would be able to form a heterodimer with RXR. Taken together, these results indicate that NR7 defines an ancient NR subfamily that arose early during metazoan evolution. Being located at a pivotal phylogenetic position, NR7 can enlighten how the heterodimerization abilities of nuclear receptors were gained in the course of evolution.

### NR7 LBD behaves as a monomer in solution

**Fig. 2:**
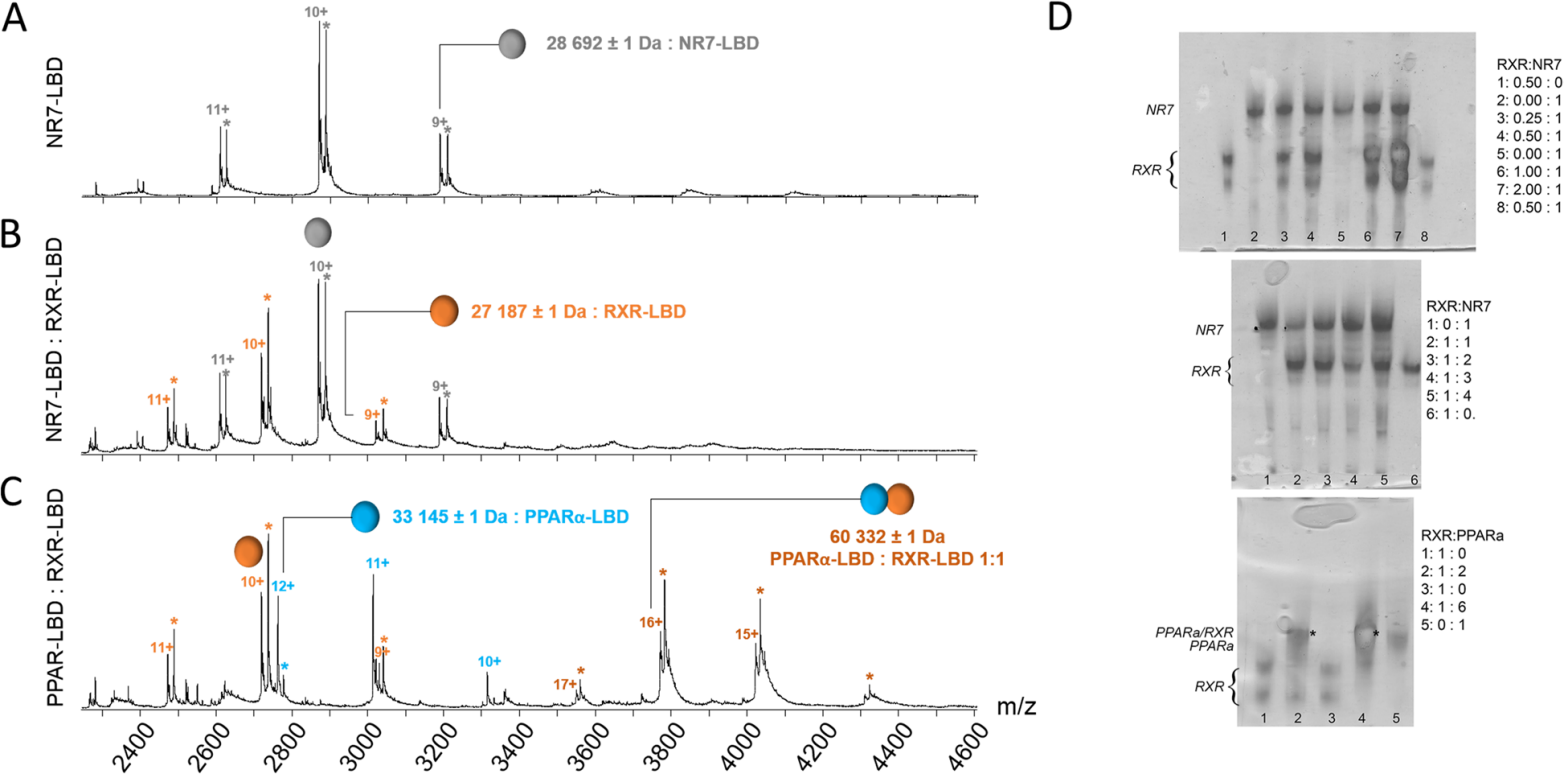
NR7 ligand-binding domain (LBD) does not heterodimerize with RXR. **A**, **B** Native mass spectrometry analysis of **A** the NR7 LBD alone and **B** the mixture NR7 LBD-RXR LBD. The different charge states of the isolated NR7 and RXR LBDs are given in grey and orange, respectively, above the m/z peaks. An α-N-6-gluconoylation modification (+178 Da, labeled with a star) of the N-terminal His_6_-tag used for protein purification is seen in a fraction of the protein, for both NR7 and RXR LBDs (grey and orange stars respectively). **C** Native mass spectrometry analysis of the human PPARα and amphioxus RXR LBDs. The different charge states of the isolated PPARα and RXR LBDs are given in blue and orange, respectively, above the m/z peaks. An α-N-6-gluconoylation modification (+178 Da, labeled with a star) of the N-terminal His_6_-tag used for protein purification is seen in a fraction of the protein, for both LBDs (given in colored stars). Peaks corresponding to the PPARα /RXR LBD complex are clearly detected (blue labels), corresponding to PPARα/RXR heterodimer formation. **D** Native polyacrylamide gel electrophoresis of amphioxus NR7 with amphioxus RXR and human PPARα and amphioxus RXR with different molar ratios indicated on the right side of the figure. Upper and middle panels: different molar ratios of RXR:NR7 were considered, by varying the quantity of RXR (upper) or NR7 (middle). No band is seen that could correspond to a heterodimer. Lower panel: different RXR:PPARα molar ratios were tested. For some RXR:PPARα ratios, an additional band (marked by a star) is observed, which corresponds to a RXR-PPARα heterodimer

Given the phylogenetical position of NR7, we investigated whether NR7 would be able to form heterodimers with RXR. We first characterized in solution the dimerization properties of its LBD that usually encompasses the main dimerization interface [2, 14]. To this end, amphioxus NR7 LBD was expressed in *E. coli*, purified to homogeneity and its oligomeric properties were characterized using native mass spectrometry (nMS) [15]. The nMS data suggest that the isolated NR7 LBD behaves mainly as a monomer (28692 ± 1 Da) (Fig. 2A, Additional file 1: Table S1).

We then asked whether the NR7 LBD has the capacity to heterodimerize with the amphioxus RXR LBD. We therefore carried out nMS measurements of NR7-RXR complex mixtures with PPARα and RXR as a positive control for heterodimerization. The results clearly indicate that heterodimerization of the NR7 LBD with the RXR LBD is not observed under our soft complex-preserving nMS measurement conditions (Fig. 2B, Additional file 1: Table S1), whereas heterodimerization of the PPARα LBD with RXR can be easily detected (60332 ± 1 Da) (Fig. 2C, Additional file 1: Table S1). The same conclusion can be drawn from native polyacrylamide gel electrophoresis experiments, where a clear band is observed for the PPARα/RXR LBD heterodimer, but not for the putative NR7/RXR dimer (Fig. 2D, Additional file 1: Table S1). Altogether, the nMS and native gel electrophoresis analyses suggest that NR7 LBD behaves as a monomer in solution.

### The NR7 LBD is a constitutive activator with a collapsed H10-H11 region

**Fig. 3:**
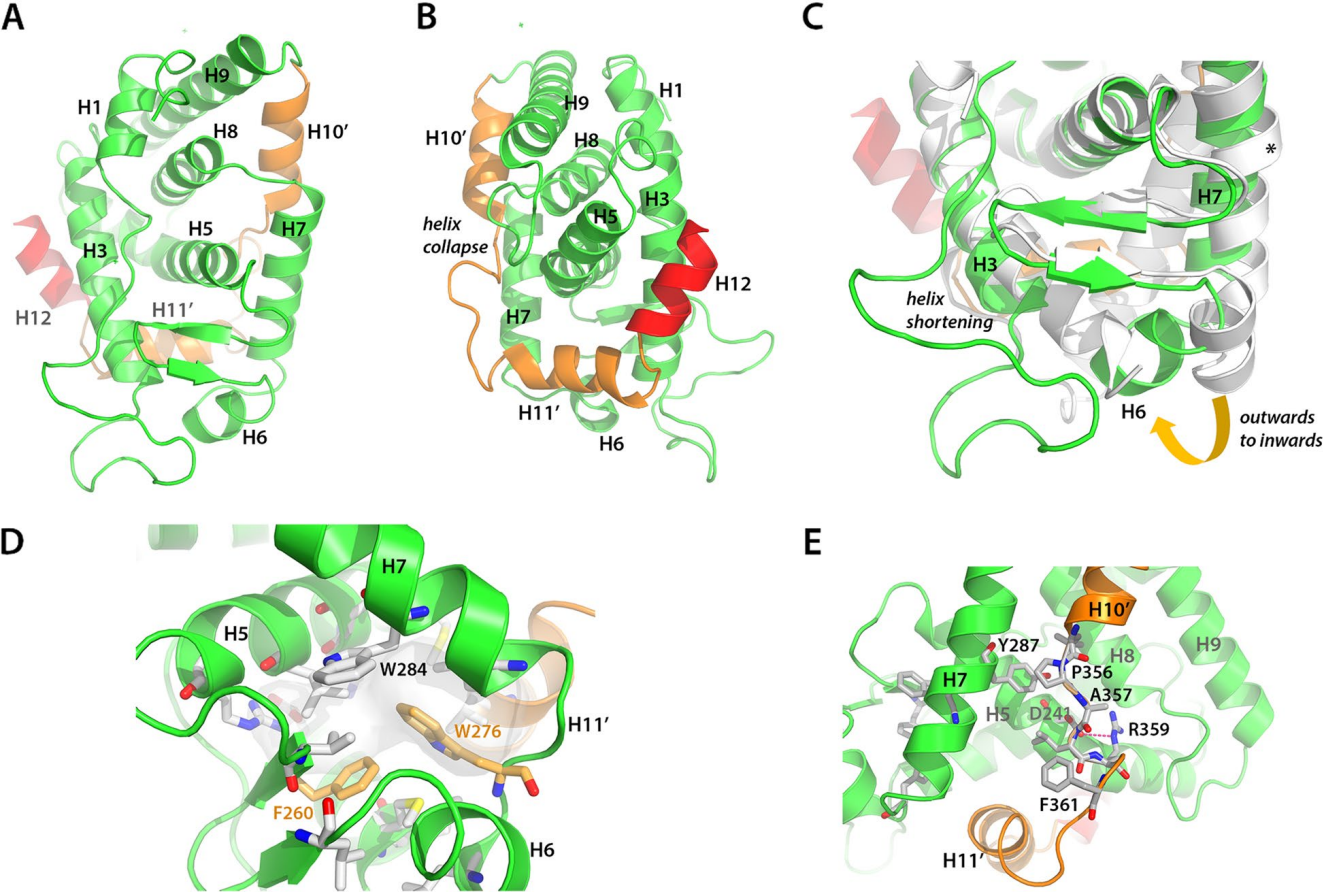
Crystal structure of the amphioxus NR7 ligand-binding domain (LBD). **A, B** Overall views from the front (**A**) and the back (**B**) of the LBD of NR7 depicted as green ribbons with corresponding helices indicated. NR7-specific features, such as the shortened helix H10 (called H10’) and the shortened helix H11 (called helix H11’) and the collapsed region connecting both helices are shown as orange ribbons. The C-terminal helix H12 is shown in red. **C** Zoom on the region of helices H3, H6, and H7 that differs markedly from the amphioxus RXR LBD structure (shown as grey ribbons), indicating a shortening of helix H3, an inwards movement of helix H6 and a straighter H7 helix as compared to RXR. The π-turn in RXR is indicated by an asterisk. **D** Enlarged view of NR7 ligand-binding pocket, showing that aromatic and hydrophobic residues fill the pocket and stabilize the apo conformation. **E** The loop H10’-H11’ makes stabilizing interactions with helix H7, with strong interactions between Phe356 and Tyr287 (H7), and with Asp241 (H5)

To uncover the structural features responsible for the monomeric behavior of the NR7 LBD, we determined its crystal structure (Fig. 3). To this end, the NR7 LBD was expressed in *E. coli*, purified to homogeneity and crystallized in P3_2_12 space group. The structure was solved using a combination of molecular replacement and anomalous diffraction from heavy atom derivatized crystals. One molecule of the NR7 LBD is found in the asymmetric unit of the crystal, suggesting that the NR7 LBD behaves as a monomer. The data collection and refinement statistics are summarized in Table 1.

**Table 1:** Native data collection and refinement statistics

	NR7
**Data collection and processing**	
Source	ESRF ID29
Detector	Pilatus 6M
Wavelength (Å)	0.9762
Resolution range (Å)	40.09–2.0 (2.07–2.0)
Space group	P3_2_12
Cell dimensions	
*a, b, c* (Å)	46.29, 46.29, 163.02
*α, β, γ* (°)	90, 90, 120
Total reflections	85020 (7025)
Unique reflections	13153 (1164)
Multiplicity	6.5 (6.0)
Completeness (%)	94.54 (85.53)
Mean *I*/σ(*I*)	23.92 (1.66)
Overall *B* factor from Wilson plot (Å^2^)	46.35
*R*_meas._ (%)	0.040 (1.051)
*R*_p.i.m._ (%)	0.015 (0.406)
CC_1/2_	1 (0.667)
CC*	1 (0.895)
**Structure refinement**	
Reflections used in refinement	13153 (1164)
Reflections used for R-free	609 (63)
*R*_work_ (%)	0.192 (0.294)
*R*_free_ (%)	0.236 (0.364)
CC(work)	0.945 (0.736)
CC(free)	0.940 (0.598)
Number of non-hydrogen atoms	1935
Macromolecules	1867
Solvent	68
Protein residues	232
R.m.s.d. from ideal values	
Bond lengths (Å)	0.014
Bond angles (°)	1.64
Ramachandran statistics	
Favored (%)	95.65
Allowed (%)	4.35
Outliers (%)	0
Average *B* factors (Å^2^)	68.7
Macromolecules	69.09
Solvent	58.03

Statistics for the highest-resolution shell are shown in parentheses

The overall structure of the NR7 LBD contains 10 α-helices arranged in a three-layered antiparallel helical sandwich with the canonical fold of NR LBDs (Fig. 3A, B). Noticeable differences compared to other NR LBDs are as follows: (*i*) the bending of helix H6 towards the interior of the receptor accompanied by the shortening of the adjacent helix H3, which is almost two turns shorter at its N-terminal end compared to what is seen in the structures of RXR or ERR LBDs (Fig. 3C) and, more remarkably, (*ii*) the collapse of the central part of the region normally attributed to helices H10 and H11 (in orange in Fig. 3B). In particular, helix 10 is shortened to a two-turn helical portion (called H10’). It collapses to an unstructured loop at the level of a proline residue that is conserved in NR7, but also found in HNF4 and RXR that is further connected to an almost perpendicularly positioned three-turn helical region that is a remnant of helix 11 (called H11’). At the C-terminal end of this short helical region is helix H12, which is located in the coactivator binding site of the LBD, hence suggesting that in the crystal, NR7 is in an inactive or repressed conformation.

The residues constituting the loop connecting H10’ and H11’ possess high B factors and weak electron density, suggesting that this region is partially disordered and flexible. In fact, the conformation of this loop is stabilized by inter-molecular interactions between crystallographic nearest neighbor molecules, in particular between residues from the loops H8-H9 and H10’-H11’ from both partners (Additional file 1: Fig. S5A). Furthermore, the bottom part of the loop H10’-H11’ adopts a peculiar bulge conformation, in which Phe361 is buried into a hydrophobic pocket formed by Leu residues of the molecule and its nearest neighbor. The conformation of this short stretch is likely stabilized in the crystal by packing interactions (Additional file 1: Fig. S5B). These observations suggest that the antagonist conformation of H12 was selected during crystallization and that H12 may nevertheless adopt an agonist conformation, as can be seen in the molecular modeling using AlphaFold [16] (see Additional file 1: Fig. S5C).

An additional feature observed in the crystal structure is the absence of a putative ligand inside the ligand-binding pocket (LBP) that could have been fortuitously trapped, as has been seen in several cases of orphan and sensor receptors [17–21]. The structural observations are further supported by nMS analysis of the purified NR7 LBD expressed in *E. coli* which demonstrates that NR7 is devoid of any fortuitously bound ligand trapped in the LBP under native conditions (Fig. 2A). The analysis of the NR7 crystal structure shows that structural elements fold into the LBP, likely contributing to the stability of the ligand-free state of the receptor (Fig. 3C). In particular, the large side chain of the aromatic residue Trp276 in the loop connecting H6 to H7 (L6-7) is inserted into the pocket and occupies a significant fraction of the cavity, leaving no space for the binding of a putative ligand (pocket size of 112 Å^3^ as calculated with CASTp, smaller than the pocket of the orphan receptor ERRα LBD) (Fig. 3D). Notice that the conformation of the AlphaFold model would still not be able to accommodate a small ligand as the pocket size does not change significantly. Other hydrophobic and aromatic residues from the bottom part of the receptor, including helices H3, H5, H6, H7, and the β-sheet further contribute to the stability of the ligand-free state, in particular Phe260 (1st β-strand) and Trp284 (H7) (Fig. 3D). These structural observations support the apo state of NR7 LBD.

To gain functional insights into the NR7 transcriptional activity, we conducted functional experiments using transient transfection assays. Amphioxus NR7 LBD was fused with the DNA-binding domain of the yeast transcription factor GAL4, and the GAL4-NR7 fusion construct was introduced into mammalian HEK293 cells with a UAS-luciferase reporter. We observed that GAL4-NR7 alone exhibits a constitutive activity that was further increased in the presence of RXR (Additional file 1: Fig. S6A). The full-length NR7 protein also activates transcription on DR4 elements in HEK293T, and this activity is increased 2.4 fold in presence of RXR (Additional file 1: Fig. S6A). This suggests that that amphioxus NR7 is a transcriptional activator capable of recruiting coactivators in mammalian cells. We further used native mass spectrometry (nMS) to characterize the binding of coactivator peptides to the *E. coli* expressed NR7 LBD that is shown to be an apo conformation, using PPARα LBD bound to the agonist GW7647 as a control receptor [22]. The nMS analysis suggests that NR7 LBD is capable of binding LXXLL containing coactivator peptides (Additional file 1: Fig. S6B), even though the interaction is rather weak compared to the control PPARα LBD (data not shown). Furthermore, mutating the two NR7 residues of the charge clamp [23], R218 in H3 and E386, to alanine, results in the significant decrease in peptide binding (Additional file 1: Fig. S6B). Note that the effects seen in the recruitment of coregulator peptides by the isolated NR7 LBD are weak and we cannot rule out that NR7 might require the DNA-induced RXR dimerization for stronger peptide binding. This would require further investigations as would be the identification of endogenous amphioxus coregulators. Finally, we observed that upon activation with a wide range of classical NR ligands, including steroids and thyroid hormones, fatty acids, and retinoids, none of these molecules was able to significantly modulate the transcriptional activity of amphioxus NR7 (Additional file 1: Fig. S6C). Altogether, these data suggest that NR7 is a constitutively active orphan receptor that functions in a ligand-independent manner.

Importantly, we notice that the dimerization interface of NR7 is peculiar, in particular with regard to the H10-H11 region. The normally long helix H10 is collapsed to a two-turn long helix, followed by a loop that connects to a short residual helix H11. The observed conformation of this region has significant consequences on the dimerization properties of NR7 and eventually on its biological function since it leads to a reduction of the dimerization interface and potentially a weak or impaired dimerization capacity. In fact, our biophysical and biochemical analyses show that the NR7 LBD does not heterodimerize with the RXR LBD, in contrast to classical heterodimeric RXR partners, such as PPARα, used as a control in our experiments. Nevertheless, we could model a canonical NR7/RXR heterodimer, if we assume that the unstructured H10’-H11’ loop region around Phe361 could change its conformation (Additional file 1: Fig. S5). In this model case, we observed that the dimerization with RXR has reduced interface area (900 Ǻ^2^) as compared to NRs that encompass a fully helical H10-H11 region (for example, 962 Ǻ^2^ for RARα/RXRα (PDB code 1DKF, as shown in Additional file 1: Fig. S7)). Thus, our analyses indicate that the peculiar conformation of the region H10-H11 of NR7 LBD is not in favor of a classical heterodimerization with RXR.

### Full NR7 dimerizes in a DNA-dependent manner

To fulfill their functions, NRs act through their LBD and DBD that represent the two functional and structural NR domains which further allosterically communicate one with the other [2]. The recognition and binding of the receptors to specific DNA sequences is essential for the transcription of NR target genes and this generally involves two hexanucleotide half-sites separated by a given spacer and arranged in a specific manner [24]. Therefore, we wondered whether full-length NR7, in contrast to its monomer behaving LBD, would act as a homo- or a heterodimer. To this end, we conducted biophysical characterization experiments, using size-exclusion chromatography coupled to native mass spectrometry (SEC-nMS) [15], SEC-MALLS and native gel electrophoresis. From the nature of key residues involved in the half-site sequence recognition (Glu56, Lys63 and Arg64 in Additional file 1: Fig. S1A), we concluded that the NR7 DBD recognizes 5′-AGGTCA-3′ sequence, like most NRs with the exception of oxosteroid nuclear receptors (*14*). We considered several typical response elements (REs), including direct repeat (DR) (DR0, DR1, DR3, DR4) and inverted repeat 3 (IR3) REs as well as a negative control DNA sequence (Ctrl(−)) (Additional file 1: Table S2). Complexes between full-length NR7 and DNA were reconstituted by mixing the purified protein with DNA with a 2 to 1 molar ratio between protein and DNA.

**Fig. 4:**
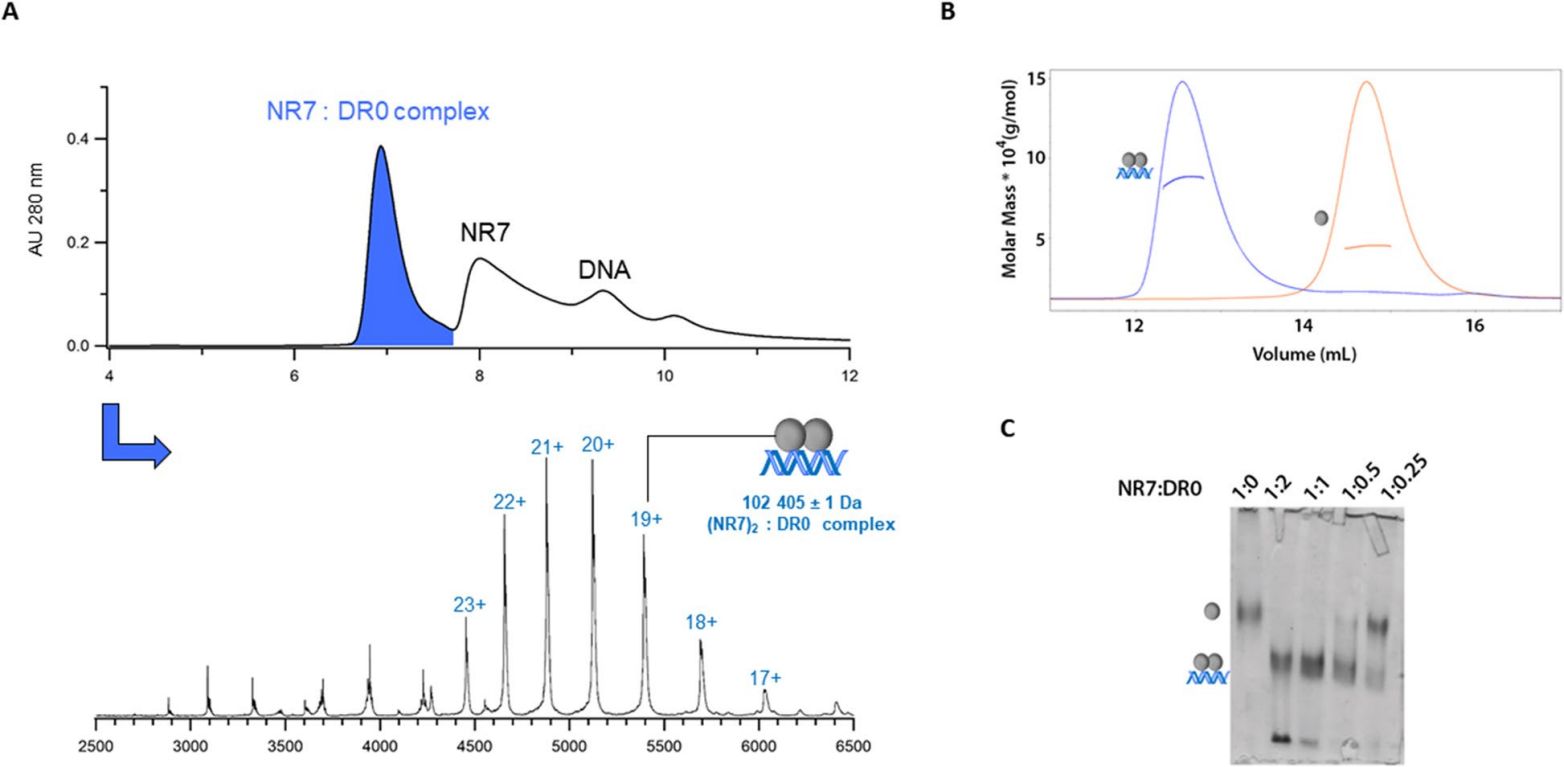
Full NR7 can homodimerize on DNA. **A** SEC-nMS analysis of full-length NR7 and DR0 response element. The upper panel corresponds to the SEC-UV chromatogram where the chromatographic peak of the homodimer of NR7 bound to DR0 is colored in blue. The lower panel corresponds to the mass spectrum extracted from the chromatographic peak colored in blue where the homodimer of NR7 bound to DR0 is identified as the main species with a mass of 102 405 ± 1 Da (charge states are given in blue.) **B** SEC-MALLS analysis of full-length NR7 in the absence and presence of DR0 response elements showing the elution profile on a SEC S200 10/300 (GE healthcare) with the direct molar mass measurement of each elution peak. NR7 elutes as a dimer when bound to DR0 with a measured molar mass of around 100 kDa and as a monomer in the absence of DNA with a measured molar mass of 43 kDa. **C** Polyacrylamide native gel of full-length NR7 with different ratios of the DR0 response element, showing the formation of an NR7:DR0 complex, as indicated on the left by the illustration

Results of SEC-nMS experiments of full-length NR7 bound to the different DNA fragments are shown in Fig. 4A for DR0 and in Additional file 1: Fig. S8 and Additional file 1: Table S3 for DR1, DR4 and Ctrl(−). The SEC-nMS analyses reveal that NR7 forms a homodimer on DR0 (102 kDa, Fig. 4A) as the main product, which is confirmed by SEC-MALLS experiments, showing that full-length NR7 is a monomer in the absence of DNA, whereas it binds DR0 as a homodimer (Fig. 4B). In addition, DNA titration assays examined with polyacryamide native gel electrophoresis demonstrate the formation of an NR7 homodimer on the DR0 response element (see Fig. 4C). Furthermore, full-length NR7 homodimerizes on other DR REs as well (Additional file 1: Fig. S8A-B), although only a minor fraction of the homodimeric species was identified with a Ctrl(−) sequence (Additional file 1: Fig. S8C), where it is instead mainly found as a monomeric DNA-unbound molecule (43 kDa). Altogether, these data demonstrate in a consistent manner that full-length NR7 remains a monomer in the absence of DNA, but that it can homodimerize on DNA.

**Fig. 5:**
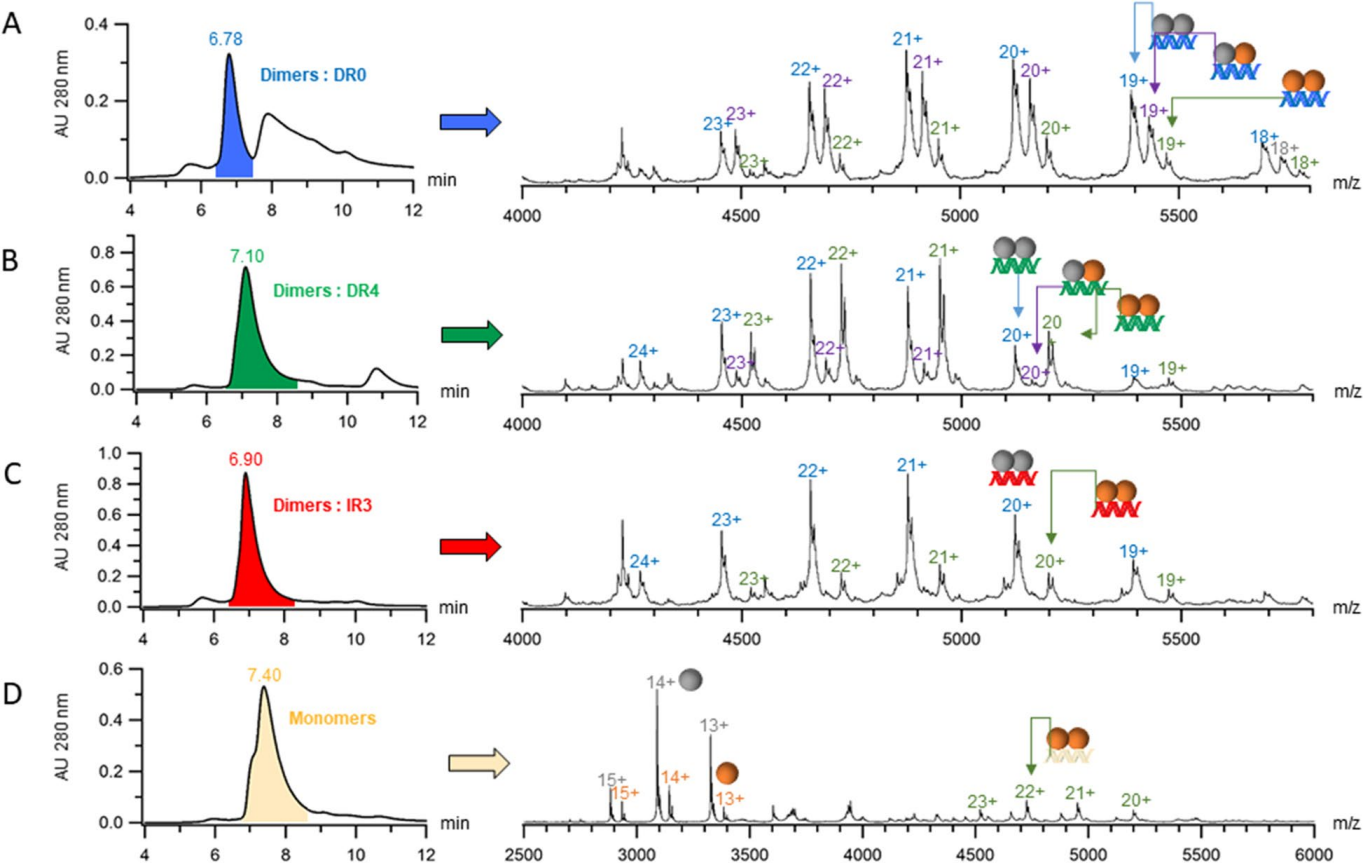
Full-length NR7 can heterodimerize with RXR on DNA. SEC-nMS analyses of full-length NR7 and RXR with **A** DR0, **B** DR4, **C** IR3, and **D** Ctrl(−) response elements (Additional file 1: Table S2). For each analysis, left panels correspond to the respective chromatograms where the main chromatographic peaks are colored in **A** blue, **B** green, **C** red, and **D** beige. Right panels correspond to the extracted mass spectra of the main chromatographic peaks. The different charge states of identified NR7 homodimers, NR7-RXR heterodimer, and RXR homodimers all bound to DNA are given in blue, purple, and green respectively while the charge states of NR7 and RXR alone are given in grey and black respectively. The masses corresponding to these identified species are summarized in Additional file 1: Table S4

Heterodimerization of full-length receptors between NR7 with RXR was next examined with SEC-nMS. As shown in Additional file 1: Fig. S9, NR7 does not heterodimerize with RXR in the absence of DNA. However, when an appropriate DNA sequence is present, NR7 and RXR are bound as a heterodimer (Fig. 5). This is indeed the case for all DR response elements considered, with a preference for DR0 and DR4, as demonstrated by the SEC-nMS analyses shown in Fig. 5A, B, Additional file 1: Fig. S10 and Table S4 and Additional file 1: Table S5. In contrast, no NR7/RXR heterodimers are observed on IR3 RE, but solely homodimers of NR7 or RXR (Fig. 5C and Additional file 1: Table S4). In addition, no heterodimers are measured on the Ctrl(−) sequence, while minor species of homodimer of RXR bound to DNA were identified (Fig. 5D and Additional file 1: Table S4), supporting the specific nature of NR7/RXR heterodimer formation on DR binding sequences. Altogether, our biophysical characterization analyses strongly suggest that DNA acts as the key modulator of the dimerization capacities of NR7, being indispensable for NR7 to heterodimerize on DR response elements such as those recognized by the classical nuclear receptor-RXR heterodimers.

### NR7 exhibits features of class I and class II nuclear receptors

**Fig. 6:**
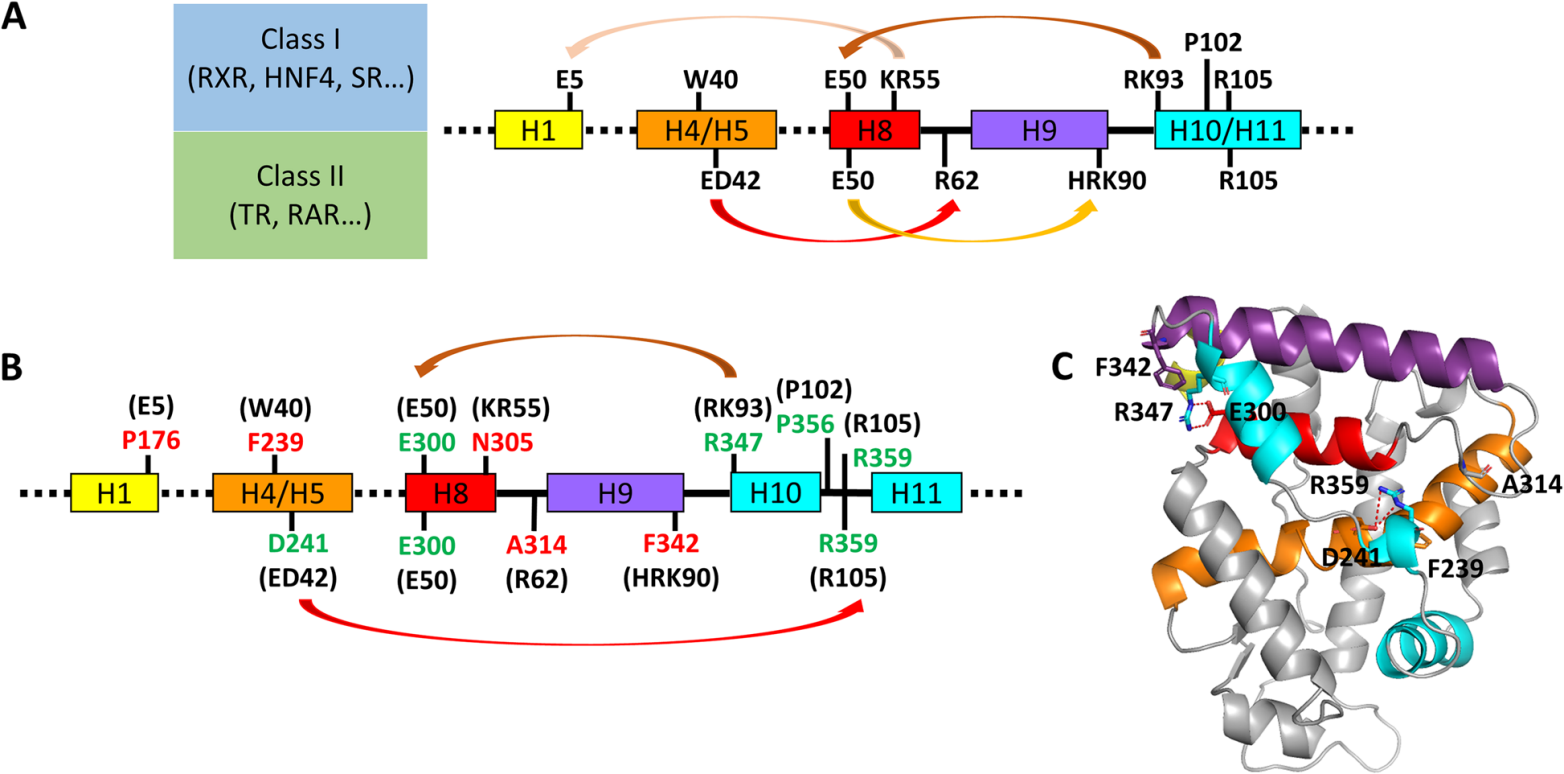
NR7-specific communication pathways. **A** Communication pathways in class I and class II nuclear receptors are depicted by arrows between class-specific residues. **B** Communication pathways in NR7. Some pathways of class I NRs are conserved in NR7, such as the aromatic residues at the junction between helices H4 and H5 and the Glu (H8) to Arg (in the loop H9-H10) interaction. Additionally, specific interactions are seen with the universally conserved arginine residue in the loop between H10 and H11 (Arg359). The numbering of residues shown in green and red correspond to the *B. lanceolatum* NR7 sequence, whereas the numbering of residues shown in black refer to the generic numbering defined previously [1]. **C** Mapping of the NR7 residues and interactions defining the communication pathways on the NR7 crystal structure shown as ribbons. The H10’-H11’ and H12 region is depicted in cyan, helices H4–H5 in orange, helix H8 in red, and helix H9 in violet

Since the structural and functional analyses indicated that the isolated NR7 LBD behaves as a monomer, we asked the question whether NR7 belongs to class I NRs that essentially encompass almost all of the monomeric and homodimeric NRs, or to class II NRs that instead include almost all NRs that form heterodimers with RXR [1]. It is known that two sets of conserved, class-specific residues in the LBD characterize class I and class II NRs. These residues form communication pathways that are intimately linked to the dimerization behavior, by connecting the dimerization region with the LBP and the coregulator binding site. Thus, their conservation represents a strong signature of their biological dimerization function. Class-specific residues and the related communication pathways were thus analyzed in NR7 sequences. The class I NR communication pathway links helix H1, which is close to the coregulator binding site, to the dimer interface through two salt bridges. The first one connects a conserved Glu in helix H1 to a Lys/Arg in helix H8, and the second one a conserved Glu in H8 to an Arg residue at the N-terminus of helix H10 (Fig. 6A, B). NR7 lacks the first salt bridge, due to the lack of the conserved Glu in H1, with no compensatory interactions. The second salt bridge between H8 and H10 is, however, well conserved (Glu300 interacting with Arg347) (Fig. 6B, C). The absence of bridging interactions between H1 and H8, as is observed in NR7, is a hallmark of class II NRs. Therefore, the presence of other class II NR specific features in NR7 was examined. Two class II-specific residues, the generic R62 (in the loop H8-H9) and HRK90 residues, are replaced by uncharged residues, A314 and F342, in NR7, making the class II-specific electrostatic interaction networks impossible in NR7 (Fig. 6B). In addition, class II NRs possess a specific salt bridge between a Glu/Asp residue at the H4-H5 kink and an Arg residue in loop L8-9 (Arg62 in generic numbering) (Fig. 6A). In NR7, the negatively charged residue Asp241 is present at the kink between H4 and H5, but rather than interacting with the Arg residue in the loop H8-H9, it makes a salt bridge with Arg359 in the loop H10’-H11’ and a stacking interaction with Tyr311 (Fig. 6B, C). Note that this NR7 Arg359 residue is highly conserved in all NRs (Arg105 in generic numbering), where it usually points towards the solvent and does not make any intra-molecular interactions. Altogether, the analysis of class-specific features suggests that NR7 possesses very peculiar communication pathways, since it has lost some class I markers, but has most of the class II markers, except the conserved Arg62. We performed an evolutionary analysis of these various amino acid positions and this reveal that the amino acids effectively change in NR7 further highlighting that NR7 represents a transition state between class I receptors and class II receptors (Additional file 1: Fig. S11).

## Discussion

### NR7 defines a new conserved NR subfamily located at a pivotal position in NR phylogeny

NR7 was initially identified in the genome of the cephalochordate amphioxus [25], but, given the paucity of genome sequences available at that time, its phylogenetic status as an independent NR subfamily could only be demonstrated in analyses based on more comprehensive datasets [7, 9, 13]. By now, NR7 orthologues have been found in the genomes of several deuterostomes, including cephalochordates, hemichordates, and echinoderms, but not in genomes of urochordates and vertebrates, suggesting that NR7 receptors were present in the last common ancestor of all deuterostomes and have secondarily been lost in urochordates and vertebrates. Similarly, NR7 is present in many, but not all protostomes. Within lophotrochozoans, genes encoding NR7 have been characterized in annelids and mollusks [13, 26] and we found an additional sequence in the brachiopod *Lingula anatina.* The presence of an NR7 in the ecdyzozoan priapulid *Priapulus caudatus* suggests parallel losses in other ecdysozoans like nematodes and arthropods. Finally, NR7 is also present in cnidarians, suggesting that this ancient NR subfamily was already present in the last common ancestor of cnidarians and bilaterians. The reason for the frequent lineage-specific losses of this gene is unclear and will require additional functional analyses. It is interesting to note however that the NR7 gene in amphioxus (Additional file 1: Fig. S3) has a conspicuous and dynamic expression pattern. The phylogenetic position of NR7 receptors is particularly striking as this subfamily is located at a key hinge in the NR tree [7, 9, 13]. Functionally speaking, NR7 is positioned at the frontier between the members of ancient NR subfamilies that bind to DNA as homodimers and the more recent NR1 and NR4 subfamilies that form heterodimers with RXR [5, 9]. Thus, NR7 represents a keystone for our understanding of the evolutionary history of the NR superfamily and the acquisition of their heterodimerization capacity.

### NR7 dimerization with RXR is DNA-mediated

The functional characterization of amphioxus NR7 shows that this receptor is capable of homodimerizing and heterodimerizing with RXR on specific DNA response elements (DR0 and DR4 in particular). However, the isolated NR7 is unable to heterodimerize with RXR in solution as demonstrated by our biochemical and biophysical characterization studies. This is consistent with the observation that the NR7 of the Pacific oyster *C. gigas* is unable to bind to RXR in a two-hybrid assay [13]. These data strongly suggest that NR7 forms a DNA-dependent NR heterodimer with RXR, in contrast to canonical heterodimers, such as TR-RXR, RAR-RXR, or LXR-RXR, that behave in a DNA-independent manner by forming heterotypic interactions between their LBDs [14]. In all these cases, the DNA is used as a platform onto which the heterodimer adapts, allowing the specific recognition of response elements in the regulatory regions of target genes. Results of our functional data are reminiscent of the behavior of the oxosteroid receptors (such as the glucocorticoid receptor and the androgen receptor), where their LBD behaves as a monomer, whereas the full receptor binds as a homodimer to specific DNA binding sites through the two DBD subunits [2].

Two observations strongly suggest that the heterodimerization pattern of NR7 is unusual. First, key elements of the crystal structure indicate that the NR7 dimerization interface is peculiar. The conformation of the C-terminal region including H10 and H11 has a shorter H10 helix and a collapsed helical region that connects to helix H11. A proline (P356) is present in H10 in the region where the helix collapses. A proline inside of a helix indeed often acts as a helix breaker, or at least creates a structural weakness. However, a proline residue is also present in RXR, HNF4, ERR, and other basal nuclear receptors. It is therefore not sufficient to explain this phenomenon. Note that the structural model predicted by AlphaFold presents a continuous helix H10-H11 consistent with what is observed for other nuclear receptors. In this case, the side chain of Phe361 is rotated inside the protein, rather than outside as seen in the crystal structure where it is stabilized by interactions with the hydrophobic environment of the residues provided by the nearest neighbor molecule. Thus, since there is probably a respiration of the protein LBD due to the absence of ligand to stabilize a unique conformation, crystal packing effects have frozen one conformation with more favorable intra-molecular interactions, especially for Phe361, that is made possible by the weakness at the level of the adjacent proline residue (Pro356).

As a consequence, a large part of the classical dimerization interface is disrupted. Yet, given the flexibility of the structure in this region, conformational changes could still create an interaction surface capable of accommodating a heterodimeric binding partner. It is therefore tempting to speculate that the interaction with DNA, by forcing the NR7 and RXR LBD in close proximity would allow this conformational change favoring stabilizing interactions with coregulators and the subsequent formation of a stable heterodimer.

**Fig. 7:**
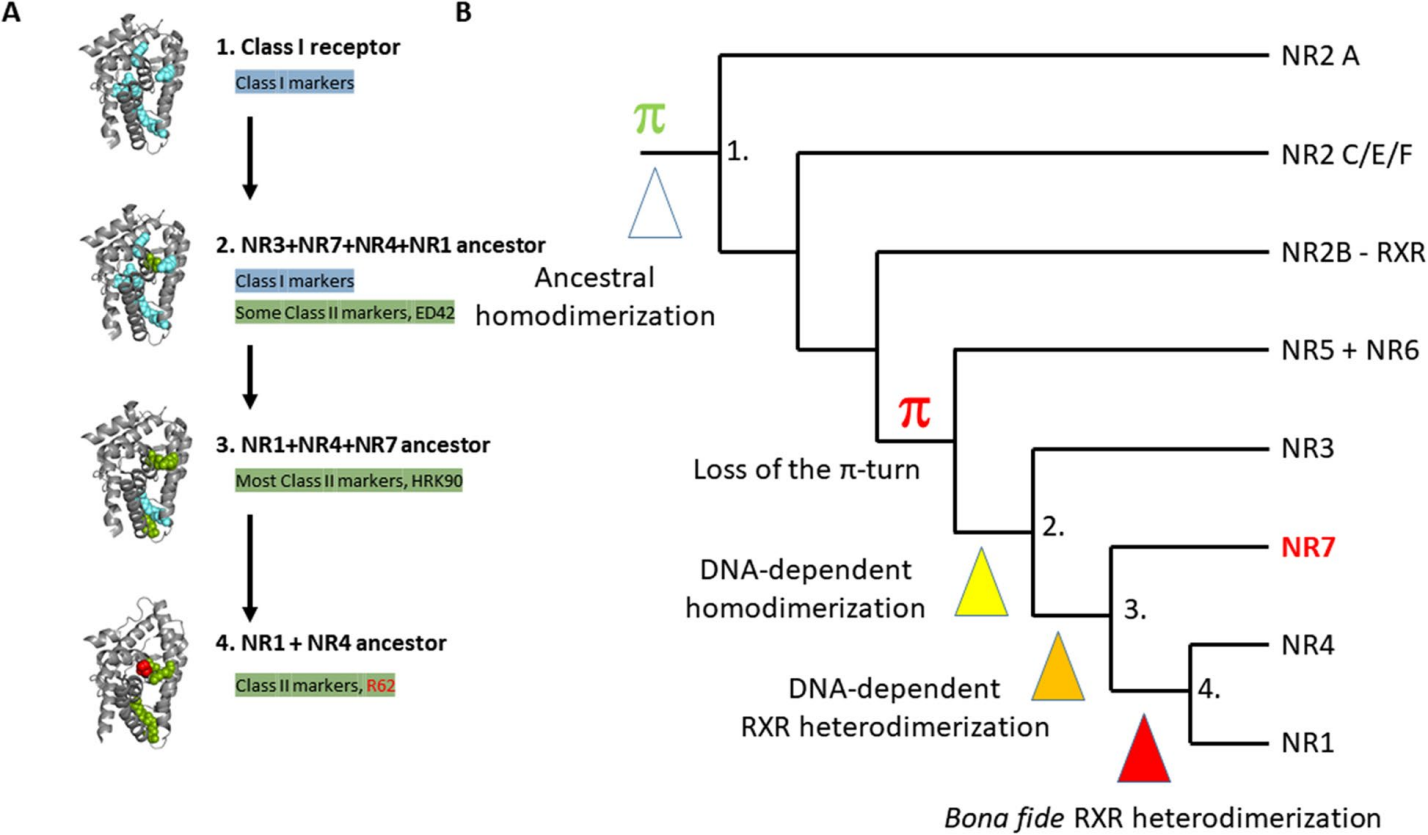
The evolution of dimerization and class I/II markers across the whole NR superfamily. **A** Successive states of class I/II markers evolution. **B** Phylogeny of NRs with the main steps in the acquisition of RXR-heterodimerization indicated. The arrows between the two panels link the state of class I/II markers with the NR diversification

Second, sequence analysis of the specific residues that distinguish between class I NRs (homodimers and monomers) and class II NRs (stable heterodimers with RXR) indicates that NR7 has a peculiar pattern of class markers, with several class I markers lost and most class II markers present (generic numbering defined in [1]). However, NR7 is not a bona fide class II receptor, since it lacks Arg62, the strictest class II marker, which is absent in all class I and present in all class II NRs. Arg62 is crucial for heterodimerization, since it forms a triad with two other class markers, the class II-specific Glu42 and the class I and II-conserved marker residue Arg105, thereby stabilizing the position of the H8-H9 loop and facilitating heterodimer interactions. We thus suggest the following scenario in which the acquisition of the bona fide class II markers resulted in a three-step process: (i) the appearance of class II markers in the NR3+NR7+NR1+NR4 and in the NR7+NR1+NR4 common ancestors, (ii) the disappearance of class I markers in the NR7+NR1+NR4 common ancestor and finally, (iii) the establishment of the Arg62 specifically in NR1+NR4 receptors (Fig. 7A). Altogether, our analysis of class-specific markers in NR7 suggests that this nuclear receptor is the “missing link” in the transition between class I nuclear receptors that were first present and class II nuclear receptors that appeared later. We propose that the functional feature underlying this paradigm is the DNA-dependency for heterodimer formation. For NR7, as for the oxosteroid receptors, DNA appears to be essential to the dimerization process. However, in contrast to oxosteroid receptors which homodimerize, NR7 heterodimerizes with RXR. How this heterodimerization activity is controlled, whether it is regulated by a putative ligand or post-translational modification and whether it exhibits a selectivity for specific types of target genes are fascinating questions that can now be explored using this unique NR.

### The evolution towards RXR heterodimers

As discussed above, NR7 is located at a key position in the NR phylogenetical tree, just between the basal class I NRs that form monomers or homodimers on direct repeats or palindromic elements, and the derived class II NRs that exhibit the classical DNA-independent RXR heterodimers (Fig. 7B). Therefore, a better understanding of the NR7 heterodimer stabilization provides novel insight into the origin and the evolution of RXR heterodimers. Dimerization of the RXR heterodimer partners at the LBD level prior to DNA binding allows an efficient flexible and specific DNA binding site recognition system. However, for NR7, the molecular structure at the level of H10/H11 hinders the formation of a strong canonical dimerization interface similar to that seen between RXR and, for example, TR, VDR, or RAR. This strongly suggests that DNA-dependent dimerization is an alternative process that is exemplified here with the NR7-RXR heterodimer. The same type of DNA-mediated dimerization requirement is observed in the oxosteroid receptors. As a consequence, the DNA-dependent dimerization implies the necessity of alternative, possibly coregulator-mediated interactions at the level of the LBDs.

## Conclusions

NR7 offers a unique outlook into the evolutionary path that links two distinct adaptive states of NR oligomerization, namely class I homodimers and class II heterodimers. Our studies combing experimental and phylogenetic analyses uncover a major issue in nuclear receptor dimerization that we show evolved from DNA-dependent to DNA-independent requirements. A deeper analysis of this new paradigm would therefore allow a better understanding of the mutational history that drives this major functional transition that paved the way to the exquisite regulatory potential of heterodimers. In particular, unraveling the influence of Arg62 and the triad of interactions formed with Glu42 and Arg105 on the H8-H9 loop rigidification and the subsequent heterodimer stabilization would allow to determine if these positions represent an epistatic ratchet, that is a series of key mutations that make changes irreversible, similar to the one described in the ligand-binding pocket of the glucocorticoid receptor [27]. It would be of major interest to unveil how this peculiar NR7 path has been selected during evolution to allow flexible heterodimeric interactions.

## Methods

### Cloning of B. lanceolatum NR7

The sequence of *B. floridae* NR7 (Table S1) was used to search available *B. lanceolatum* transcriptome databases [28]. The resulting *B. lanceolatum* sequences allowed the design of *B. lanceolatum* NR7-specific primers (Forward: 5′-TAAACAACATGGCGAGACA-3′; Reverse: 5′-ATGACACCTTAGTCAAAGCA-3′). Total mRNA was extracted from *B. lanceolatum* adults with the RNeasy Minikit (Qiagen) and was used as template for cDNA synthesis using the Superscript III kit (Invitrogen) and random primers. Subsequent PCR experiments were performed using AmpliTaq Gold (Roche), and amplified fragments were cloned using the pGEM-T Easy system (Promega) before being sequenced on both strands. The resulting NR7 sequence from *B. lanceolatum* is 1232 bp long and partially truncated at its N-terminus. The *B. lanceolatum* NR7 clone thus contains the C-terminal portion of the A/B domain, complete DBD, hinge, and LBD domains as well as the STOP codon.

Several constructs were cloned specifically for the structural and biophysical experiments: *B. lanceolatum* NR7 LBD (amino acids Ser169 to Ser389 were cloned into the in-house pET-derived expression vector, pnEAtH), *B. lanceolatum* NR7 ΔA/B construct (amino acids 30 to 389 were cloned into a pET15b expression vector). Because of incomplete available sequence of the full *B. lanceolatum* NR7, amino acids are numbered according to the available sequence to date which is given in Suppl. Fig. S1A. *B. lanceolatum* RXR ΔA/B construct (amino acids 151 to 522) was cloned into a pET15b expression vector. Expression of the different proteins cloned in these vectors was carried out in *E. coli* as specified below.

### Phylogenetic analyses

Our backbone dataset for the extensive outgroup was simplified from [7]. Additional sequences for the NR7 subfamily were retrieved by BLAST against metazoan proteins. Protein sequences were aligned with Muscle [29], and alignments were checked by eye and edited with Seaview [30]. Phylogenetic trees were calculated using PHYML [31], a fast and accurate maximum likelihood method, under the LG substitution model [32]. Node robustness was assessed using approximate likelihood-ratio test (aLRT) [33]. Accession numbers are provided in Additional file 1: Data S1.

### Amphioxus collection and spawning

Ripe adults of the European amphioxus, *B. lanceolatum*, were collected near Argelès-sur-Mer, France, and maintained in an artificial sea water facility at 19°C under natural light and dark conditions [34, 35]. Spawning was induced with a thermal shock (from 19 to 23°C) as previously described [34, 35]. In vitro fertilization was performed and embryos and larvae were collected at different stages of development [34, 35].

### Whole mount in situ hybridization

For amphioxus, linearized plasmids were used as templates for synthesizing riboprobes using the DIG labeling system following manufacturer’s instructions (Roche). DIG-labeled riboprobes were purified using lithium chloride precipitation, dissolved in 50% formamide at 100 ng/μl, checked on an agarose gel, and stored at −20°C. Embryos and larvae were fixed overnight at 4°C in 4% paraformaldehyde (PFA) in 0.1 M MOPS, 2 mM MgSO_4_, 1 mM EGTA, 0.5 M NaCl, pH 7.5. Fixed embryos and larvae were then washed and stored in 70% ethanol at −20°C. The in situ hybridization protocol is described in [36].

### Amphioxus quantitative PCR (qPCR) analyses

We used the qPCR method described in [36]. Amphioxus embryos were collected at different stages, frozen in liquid nitrogen, and conserved at −80°C. Adult amphioxus were collected and conserved in RNAlater (Qiagen) at −20°C. A Precellys homogenizer (Ozyme) was used to homogenize the amphioxus material and total RNAs were extracted using an RNeasy kit (Qiagen) following the manufacturer’s instructions. Purity of extracted RNAs was analyzed using a DropsSense spectrophotometer (Trinean). RNAs were then treated with TurboDNAse (Ambion) following the manufacturer’s instructions. The concentration of DNAse-treated RNAs was analyzed with a NanoDrop 2000 spectrophotometer (Thermo Fisher Scientific), and the quality of RNA samples was analyzed using a TapeStation (Agilent). Because of the difficulty of obtaining significant amounts of embryonic material from amphioxus, only one RNA extraction was performed per condition. RNA samples with RINe>7.8 were retained for reverse transcription. Two hundred nanograms of embryonic RNA and 500 ng of adult RNA were used for NR7 expression analyses. RNAs were reverse transcribed with High Capacity RNA-to-cDNA reverse transcriptase (Applied Biosystems) using a mixture of random hexamers and oligodT primers following the manufacturer’s instructions. Primers were designed using Primer3Plus (http://www.bioinformatics.nl/cgi-bin/primer3plus/primer3plus.cgi/). The qPCR reactions were performed following the manufacturer’s instructions with IQ SYBR Green Supermix (Biorad) on a CFX96 Real-Time PCR Detection System (Biorad). The reaction conditions were as follows: 95°C for 3 min followed by 45 cycles of 95°C for 10 s and 60°C for 30 s. Data were collected and curves were generated with the CFX Manager software (Biorad). Each reaction was carried out in technical triplicates. Dissociation analysis was performed at the end of each reaction to confirm the amplification specificity. For each reaction, a reverse transcriptase negative control was performed to test for genomic DNA contamination. To determine primer efficiencies, a template qPCR reaction was performed on adult cDNA for each primer couple and then diluted (with seven dilution points) to generate linear standard curves.

### Transactivation assays

Human embryonic kidney 293 cells were grown in Dulbecco’s modified Eagle’s medium supplemented with 10% coal-stripped fetal bovine serum and penicillin/streptomycin at 100 mg/ml. Cells were maintained at 37°C with 5% CO_2_. Transient transfection assays were carried out in 96-well plate with 30 000 cells per well using the Exgen 500 transfection agent (Thermo Fisher Scientific) according to the manufacturer’s instructions. For each well, cells were transfected with 50 ng of total DNA: using 12.5 ng of GAL4-NR7(LBD) plasmid, 12.5 ng of UAS-Luciferase reporter plasmid, 12.5 ng of β-galactosidase plasmid, and 12.5 ng of pG4M empty plasmid or amphioxus RXR plasmid for the GAL4/UAS experiments and 12.5 ng of NR7 full-length plasmid, 12.5 ng of DR4-Luciferase reporter plasmid, 12.5 ng of β-galactosidase plasmid, and 12.5 ng of pG4M empty plasmid or amphioxus RXR plasmid for the full-length/DR4 experiment. Transfected cells were incubated for 48 h with or without drug and harvested using a passive lysis buffer and frozen at −20°C.

Luciferase activities were assayed with the luciferase reagent buffer (Promega) on a Veritas luminometer (Turner BioSystems). The β-galactosidase activity was measured using ONPG substrate and absorbance at 420 nm as internal standardization. Each assay was performed at least three times independently on well triplicates. Drugs purchased from Sigma-Aldrich were first diluted in DMSO or ethanol 100% according to the manufacturer recommendation at 10^−2^ M then in sterile PBS 1× at 10^−3^ M prior treatment.

### Cloning, expression, and purification for structural studies

The expression vectors for the different protein constructs were transformed separately into *Escherichia coli* BL21 (DE3). Cells were grown at 37°C and induced for protein expression at an OD_600nm_ of 0.6 with 1 mM IPTG at 25°C for 3 h. The cell pellet was resuspended in binding buffer (20 mM Tris pH=8.0, 400 mM NaCl, 10 % glycerol, 2 mM CHAPS, 5 mM imidazole) and lysed by sonication. The crude extract was centrifuged at 45,000*g* for 1 h at 4°C. The lysate was loaded on a Ni affinity step on HisTrap FF crude column (GE Healthcare, Inc.), and the protein was eluted at a concentration of 150 mM imidazole. The hexahistidine tag was cleaved overnight using thrombin protease. The different proteins were then polished by size-exclusion chromatography in a SEC buffer (10 mM Tris pH=8.0, 250 mM NaCl, 2 mM CHAPS) by using a Superdex S75 (LBD) or S200 (full) 16/60 column (GE Healthcare).

### Crystallization

The NR7 LBD was concentrated to 9.6 mg/ml. Crystallization experiments were carried out by sitting drop vapor diffusion at 293K using a Mosquito Crystal nanoliter dispensing robot (SPT Labtech). Equal volumes (200 nl) of the protein and the reservoir solution were mixed and equilibrated against 40 μl of reservoir solution in 96-well 3-drop MRC crystallization plates (Molecular Dimensions), using both commercial (Qiagen, Hampton Research, Molecular Dimensions and Rigaku Reagents) and in-house crystallization screens. Diffraction quality crystals were obtained in 1.3 M Li_2_SO_4_, 0.1 M HEPES pH=7.5. Crystals were equilibrated in a stepwise manner by increasing the concentration of Li_2_SO_4_ to 2 M and then flash cooled in liquid nitrogen.

### Data collection and phasing

Native datasets were collected to a maximum resolution of 2 Å on the ID29 beamline at the European Synchrotron Radiation Facility (ESRF). Data from thiomersal derivatized crystals were collected on the X06DA beamline at the Swiss Light Source (SLS). All data were integrated, indexed, and scaled using XDS [37]. The crystals showed a primitive trigonal space group, either P3_1_12 or P3_2_12 with unit cell dimensions a=b= 46.4 Å and c=163.2 Å. Since amphioxus NR7 does not have direct homologs in model organisms, such as vertebrates, molecular replacement was attempted with several closely related crystal structures, in particular with structures of the RXR LBD with which NR7 has 46 % sequence identity. Initial models were prepared by removing flexible loops and known variable regions such as the β-sheet and the C-terminal helix. All of the models, except one, failed to give a reasonable MR solution. A weak solution was found in P3_2_12 using the mollusk RXR (PDB code 1XIU) with one molecule per asymmetric unit with reasonable packing, using Phaser [38] in the PHENIX suite [34]. Alternatively, a similar solution was obtained using the BALBES software [39]. However, the maps were very poor, especially for the lower part of the protein, including helices H6, H7, H10, and H11 and the construction/refinement of the structure stalled at an early stage with no further improvement in the map quality. Therefore, soaking of the crystals with several different types of heavy atom (HA) compounds was undertaken. The best result was obtained by soaking the crystals in a thiomersal solution at a concentration of 1 mM for 24 h. Two HA sites were found using AutoSHARP [40, 41] in the CCP4 suite [42]. However, the maps were poor and it was not possible to build a model. The partial molecular replacement solution was refined against this derivative dataset which allowed the identification of the mercury sites (one fully occupied dual conformation site and two partially occupied sites (with about 25% occupancy), and placement of the mercury atoms vastly improved the map quality.

### Model building and refinement

Refinement was performed using Refine in the PHENIX suite and BUSTER [43], followed by iterative cycles of construction in COOT [44]. Once the structure had been refined to convergence, it was refined against the best native dataset. Figures were prepared using PyMOL version 1.7.4.0 (Schrödinger, LLC, [45]) and the interface area was calculated using CoCoMaps software [46]. The coordinates and associated structure factors have been deposited in the Protein Data Bank under the accession code 7Q71.

### Native polyacrylamide gel electrophoresis

Oligonucleotides (Additional file 1: Table S2) were annealed at 1 mM in 10 mM Tris HCl pH 8.0, 100 mM NaCl, and 0.1 mM EDTA, and incubated with purified recombinant NR7 and RXR in a 1:1.2 protein dimer: DNA molar ratio as described [47]. The protein complexes were run on an 8% polyacrylamide gel (PAGE) at 2 W constant power after pre-running the gel for 40 min at 4°C. The native gel system was based on a Tris/CAPS (3-cyclohexylamino-1-propanesulfonic acid) (pH 9.4) buffer system that contained 60 mM Tris base and 40 mM CAPS. Approximately 3 to 5 μg protein was loaded per lane along with its DNA counterpart at defined molar ratios. The polyacrylamide gels were stained using Instant Blue Protein Stain (Expedeon Protein Solutions) for 15 min and subsequently rinsed in water.

### Native mass spectrometry (nMS)

Native Electrospray-Mass Spectrometry (ESI-MS) analyses were performed on an electrospray quadrupole-time-of-flight mass spectrometer (Synapt G2 HDMS, Waters, Manchester, UK). The mass spectrometer was calibrated using singly charged ions produced by a 2 g/L solution of cesium iodide (Acros organics, Thermo Fisher Scientific, Waltham, MA USA) in 2-propanol/water (50/50 v/v). Prior to injection, samples were buffer exchanged in 150 mM ammonium acetate (NH_4_OAc), pH 7.0 buffer using 0.5 mL Zeba^TM^ Spin desalting columns (Thermo Fisher Scientific, Waltham, MA USA). After buffer exchange, concentrations were determined by UV-Vis using a Nanodrop 2000 Spectrophotometer (Thermo Fisher Scientific, Waltham, MA USA). Samples were diluted in pH 7.1, 150 mM ammonium acetate buffer and mixed as follows: NR7 LBD alone was analyzed at 5μM; NR7 LBD and RXR LBD molecules were mixed with a molar ratio of 1:2 (5 and 10 μM respectively); PPARα LBD and RXR LBD molecules were also mixed with a molar ratio of 1:2 (5 and 10 μM respectively). Samples were then infused into the mass spectrometer via an automated chip-based nanoESI source (Triversa Nanomate, Advion, Ithaca, NY) with capillary voltage and gas pressure set to 1.65 kV and 0.65 psi respectively. Instrumental parameters of the mass spectrometer were optimized for the detection of labile noncovalent complexes as follows: interface pressure, 6 mbar; cone voltage, 80 V (for mixture analyses), and 120 V (for NR7 LBD alone analysis); m/z range, 1000–10,000; scan time, 4 s. Native MS data treatment was performed using Mass Lynx V4.1 (Waters, Manchester, UK). Deconvolution was performed using UniDEc [48]. Relative abundances of the species were calculated from native MS intensities of the deconvoluted data.

### SEC coupled to native MS (SEC-nMS)

For SEC-nMS, an ACQUITY UPLC H-class system (Waters, Manchester, UK) comprising a quaternary solvent manager, a sample manager cooled at 10°C, a column oven at ambient temperature, and a TUV detector operating at 280 and 260 nm was coupled to the Synapt G2 HDMS mass spectrometer (Waters, Manchester, UK). All analyses were realized with a baseline response element concentration of 5 μM. Additionally, NR7:RXR:DNA analyses involved 10μM of each protein while single protein:DNA analyses involved 20μM of NR7 or RXR. The different samples were loaded (25 μL) on an ACQUITY UPLC Protein BEH SEC column (4.6 × 150 mm, 1.7 μm particle size, 200 Å pore size from Waters, Manchester, UK) using an isocratic elution with pH 6.8, 150 mM ammonium acetate solvent at a flow rate of 0.25 mL/min over 3 min. The flow rate was then decreased to 0.10 mL/min over 10 min and finally increased to 0.25 mL/min over 5 min. The Synapt G2 HDMS was operated in positive ion mode and instrumental parameters were optimized as follows: capillary voltage, 3 kV; interface pressure, 6 mbar; cone voltage, 180 V; m/z range, 1000–10,000; scan time, 1.5 s. SEC-nMS data interpretations were performed using Mass Lynx V4.1 (Waters, Manchester, UK).

### Size-exclusion chromatography coupled to multi-angle light scattering

SEC-MALS/QELS experiments were performed on a multi-angle light scattering detector (miniDAWN TREOS, Wyatt Technologies) coupled in-line with SEC and an interferometric refractometer (Optilab T-rEX, Wyatt Technologies). A Superdex S200 10/300 GL column (total volume 24 mL, GE Healthcare) with a flow rate of 0.5 mL/min was used to separate the sample before performing the MALLS/QELS measurement. Experiments were done with 50–100 μL samples at concentrations between 1 and 3 mg/mL in a buffer of composition 20 mM Tris pH 8, 120 mM NaCl; 2 mM MgCl_2_ and 2mM TCEP. The molar mass was determined by construction of Debye plot using Zimm formalism (plot of K*c/R(θ) as a function of sin^2^(θ/2)) at 1-s data interval. The analysis of the data was performed using the ASTRA 6.1software (Wyatt Technologies).

## Supplementary Information

**Additional file 1: Fig. S1.** Sequence of amphioxus NR7. **(A**) Amphioxus (*Branchiostoma lanceolatum*) NR7 nucleotide sequence with the corresponding amino acid translation. **(B**) Comparison of amphioxus NR7 with NR7 sequences from other species. The DNA-binding domain (DBD) (upper panel) and ligand-binding domain (LBD) (lower panel) are shown. **(C**) Alignment of the known sequence of *Branchiostoma lanceolatum* NR7 (1-389) with the sequence of *Branchiostoma floridae* NR7 (1-425). Sequence conservation is indicated at the bottom. Above the sequence is the LBD helix representation of the crystallographic structure and the structure predicted with AlphaFold. **Fig. S2.** Phylogenetic analysis of the nuclear receptor (NR) superfamily. The maximum likelihood tree corresponds to the shortened version presented Fig. 1. Classical NR subfamilies are simplified as triangles. Branch support values were assessed by approximate likelihood-ratio test (aLRT) and are plotted only if superior to 0.97, which is considered fully robust. Accession numbers are given in the Additional file 1: Data S1. **Fig. S3.** Developmental expression of amphioxus (*Branchiostoma lanceolatum*) NR7 established by whole mount *in situ* hybridization. Maternal expression of NR7 is detectable at the 8-cell stage (**A**) and remains detectable at blastula stages (**B**). At the gastrula stage (**C**), NR7 expression is in the anterior ectoderm (black arrow). Dorsal (**D**) and lateral (**E**) views of an early neurula. (**F**) Lateral view of a mid neurula. NR7 is expressed in the endoderm. (**G**) Late neurula in lateral view with NR7 expression in the cerebral vesicle of the anterior central nervous system, the gut endodern and the club-shaped gland in the pharynx. (**H**) Higher magnification of the region outlined in (**G**). Black arrow marks the signal in the cerebral vesicle and the arrowhead points to expression in the club-shaped gland. (**I**) Lateral view of a larva. (**J-K**): Higher magnification of the region outlined in (**I**). (**J**) Focus on the pharyngeal region of the larva, with white arrowhead pointing to NR7 expression in the club-shaped gland. (**K**) Focus on the central nervous system, with black arrowhead highlighting expression in the cerebral vesicle and black arrow indicating expression in the pre-oral pit. Scale bars are 100 μm. **Fig. S4.** Mass spectrometric analysis of amphioxus NR7. (A). Native mass spectrometric analysis of NR7 indicating that the NR7 LBD has been purified in the apo form, without any fortuitous ligand being trapped inside the ligand-binding pocket, as shown by the grey dots above the m/z peaks. A α-N-6-phosphogluconoylation modification of the N-terminal His_6_-tag used for protein purification is also observed and corresponds to a second series of m/z peaks, as indicated by black dots above the m/z peaks. The NR7 LBD behaves essentially like a monomer, with only a very small fraction of dimer observed. (B-D). Size-exclusion chromatography (SEC)-coupled native mass spectrometric analysis of NR7 LBD. In (B) the totality of the SEC peak shown in the upper panel was integrated and the mass spectrum is shown in the lower panel. The results indicate that the dominant fraction of NR7 and of the NR7 modified by α-N-6-phosphogluconoylation His_6_-tag are monomeric, with a very minor amount of homodimeric species. The same symbols are used as in (A). (C) and (D) show mass spectra of the first and the last fractions of the SEC peaks, respectively, with the SEC peak shown in the insert. Monomeric NR7 is seen at the beginning (C) as well as in the end (D) of the SEC peak. In contrast, the small fraction of dimeric NR7 species is observed mostly at the end of the peak (D), which is not consistent with the classical behavior of multimeric particles on SEC columns, where larger species appear at earlier retention times than smaller ones. This strongly suggests that dimers are formed during the ionization process of the MS analysis and thus represent an ionization artifact. **Fig. S5.** Crystal packing effects stabilize the conformation of the loop connecting helix H10’ to H11’ in amphioxus NR7. **(A**) Ribbon representation of the amphioxus NR7 ligand-binding domain (LBD) (green) with the crystallographic nearest neighbor (grey) that stabilizes the H10’-H11’ loop conformation. Corresponding helices are indicated. NR7-specific features, such as the shorter helices H10 (H10’) and H11 (H11’) are shown in orange and the collapsed region connecting the two helices is shown as a yellow ribbon. The C-terminal helix H12 is shown in red. The nearest neighbor is shown in grey, and its C-terminal helix H12 is in dark red. **(B**) Zoom on the interaction region between the H10’-H11’ loop of NR7 and its nearest neighbor. The aromatic residue Phe361 of this loop is buried into a hydrophobic pocket formed by leucine residues of the nearest neighbor, stabilizing a peculiar bulge conformation at this location. **(C)** Comparison of the crystal structure of NR7 LBD (green) with the structure predicted by AlphaFold colab (cyan). AlphaFold predicts that NR7 can nevertheless adopt an agonist H12 conformation with a long, continuous H10-H11 helix. The orange arrow indicates that the small H11 helical part in the crystal structure would interferes with an agonist position of helix H12 as observed in the AlphaFold model. **Fig. S6.** Functional characterization of amphioxus NR7. **(A**) Transactivation assay in HEK293T cells of Gal4-NR7 LBD with a UAS-luciferase reporter. Shown are the results for transfection of the control plasmid (UAS), Gal4-NR7 LBD, Gal4-NR7 LBD plus RXR relative to the control UAS (left panel), as well as NR7 full-length on DR4, NR7 full-length plus RXR on DR4 relative to control (DR4 alone, right panel). Error bars show the standard deviation of the mean. **(B**) Mass spectrometric analysis (nMS) of LxxLL coregulator peptide binding to NR7 wild type (wt) and to R218A+E386A mutant NR7 LBD. In i) and ii) are shown mass spectra obtained by deconvolution of the raw data for NR7 wt (i) and mutant (ii) in complex with the PGC-1A LxxLL peptide (molar ration 1:3). iii) Quantification of the species from the mass spectra, shown as histograms, of the relative abundance (%) for the different LxxLL containing peptides shown in iv) with NR7 LBD wt (dark grey) and NR7 LBD R218A+E386A mutant (light grey). In iv) the experimental masses are given for a cone voltage set at 60 volts. “nd” means “not detected”. (**C**) Transactivation assay in HEK293T cells of Gal4-NR7 LBD with a UAS-luciferase reporter in the presence of different nuclear receptor ligands at a final concentration of 10^-5^M. The ratios of treatments over controls (GAL4-MH construct) are shown. The error bars show the standard deviation of the mean. no treatment: control; T4, T3, Triac: different thyroid hormone derivatives; NH3: thyroid hormone receptor antagonist; DHA: docosahexaenoic acid; β-carot: β-carotene; ATRA: all-*trans* retinoic acid; 9cRA: 9-*cis* retinoic acid; GW: GW409544, a PPARα agonist; T0: T0901317, a LXRα agonist; 20H-Ecd: 20-hydroxyecdysone; BPA: bisphenol A; TBBPA: tetrabromobisphenol A, ICI: ICI1872780, an ER antagonist; E2: 17β-estradiol; OHT: 4-hydroxytamoxifen; R1881: metribolone. **Fig. S7.** Heterodimer formation of amphioxus NR7 and RXR. The ligand-binding domains (LBDs) of amphioxus NR7 and RXR create a reduced interface area compared to classical RXR heterodimers. Views from the front and rotated by 90° of a model of amphioxus NR7-RXR (on the left) built by superimposing the crystal structure of the isolated proteins to the human RAR-RXR heterodimer (PDB code 1DKF) (on the right). The loop between helices H10’-H11’ is removed from the NR7 structure because of serious steric clashes with RXR and is further considered for interface area calculations. The short helix H10’ and helix H12 of NR7 are shown in orange and red, respectively. **Fig. S8.** Full NR7 forms a homodimer on direct repeat response elements, but not on control DNA. SEC-nMS analyses of full NR7 with (**A**) DR1, (**B**) DR4 and (**C**) Ctrl(-) response elements. For each analysis, left panels correspond to the respective chromatograms where the main chromatographic peaks are colored in (**A**) pink, (**B**) green and (**C**) beige. Right panels correspond to the extracted mass spectra of the main chromatographic peaks. The different charge states of identified NR7 bound to DNA as monomers or homodimers are given in black and blue respectively while charge states of full NR7 alone are given in grey. The masses corresponding to these identified species are summarized in Additional file 1: Table S3. **Fig. S9.** Full NR7 does not heterodimerize with RXR in absence of DNA. SEC-nMS analysis of full NR7 and RXR. The upper panel corresponds the SEC-UV chromatogram where the chromatographic peaks corresponding to RXR and NR7 are colored in orange and grey respectively. The lower panels correspond to the mass spectra extracted from the chromatographic peaks colored in orange and grey (spectrum ① and ② respectively). In these spectra, full NR7 (grey charge states) and RXR (orange charge states) are identified as monomers with a mass of 43235 ± 1 Da and 44002 ± 1 Da respectively. **Fig. S10.** Full NR7 heterodimerizes with RXR on other AGGTCA response elements organized as DR1 and DR3. SEC-nMS analyses of full NR7 and RXR with (**A**) DR1 and (**B**) DR3 response elements. For each analysis, left panels correspond to the respective chromatograms where the main chromatographic peaks are colored in (**A**) pink (A) and (**B**) dark blue. Right panels correspond to the extracted mass spectra of the main chromatographic peaks. The different charge states of identified NR7 homodimers, NR7-RXR heterodimer and RXR homodimers all bound to DNA are given in blue, purple and green respectively. The masses corresponding to these identified species are summarized in Additional file 1: Table S5. **Fig. S11.** Evolutionary analysis of critical class I and class II amino acids residues involved in dimerization, plus the destabilizing proline 102. **A.** Schematic representation of the positions of class I- and class II-specific residues plus Pro 102. Class-specific residues are also indicated on a 3D structure. **B.** Mapping of some critical residues on a simplified phylogenetic tree of the NR family, using the procedure described in Beinsteiner et al., 2021. **Table S1.** Theoretical masses and measured masses of the identified species relative to SEC-nMS analyses of the NR7 LBD, RXR LBD and PPAR LBD, alone or in mixture. The star relates to the observed α-N-6-phosphogluconoylation (+178 Da) modification of the N-terminal His_6_-tag. **Table S2.** Oligonucleotides used for polyacrylamide gel electrophoresis experiments. Half-sites of the REs are underlined. **Table S3.** Measured masses of the identified species relative to SEC-nMS analyses of full NR7 with DR1, DR4 and Ctrl(-) response elements (Additional file 1: Fig. S8). Masses corresponding to full NR7 alone or bound to DNA as monomer or homodimer are reported in this table. **Table S4.** Measured masses of the identified species relative to SEC-nMS analyses of full NR7 with DR0, DR4, IR3 and Ctrl(-) response elements (**Fig.** **5**). Masses corresponding to full NR7, RXR alone, NR7 homodimers, NR7-RXR heterodimer and RXR homodimers all bound to DNA are reported in this table (n.d. : not detected). **Table S5.** Measured masses of the identified species relative to SEC-nMS analyses of full NR7 with DR1 and DR3 response elements (as shown in Additional file 1: Fig. S10). **Data S1.** Accession number for sequences used in the tree of Additional file 1: Fig. S1. Accession numbers are coming mainly from GenBank, and alternatively from UniProt or from the website of the Joint Institute of Genomics (JGI). The four species for which the sequences are coming from the JGI can be accessed using the following species-specific search pages: https://genome.jgi.doe.gov/pages/search-for-genes.jsf?organism=Capca1, https://genome.jgi.doe.gov/pages/search-for-genes.jsf?organism=Helro1, https://genome.jgi.doe.gov/pages/search-for-genes.jsf?organism=Lotgi1, https://genome.jgi.doe.gov/pages/search-for-genes.jsf?organism=Nemve1

## Data Availability

*GenBank accession number*: The sequence data have been deposited to GenBank. Amphioxus NR7: MK976734, sea urchin NR7: MK976735 and leech NR7: XP_009028197.1 *Protein Data Bank accession number*: The crystal structure of NR7 LBD has been deposited to the Protein data Bank (www.pdb.org) under the accession number 7Q71.
