# Supplementary material for: A novel nuclear receptor subfamily enlightens the origin of heterodimerization
Source: BMC Biol. 2022 Oct 5;20:217. doi: 10.1186/s12915-022-01413-0 (PMC9535869; doi:10.1186/s12915-022-01413-0)
Supplement: Supplementary file 1 — Additional file 1: Fig. S1. Sequence of amphioxus NR7. (A) Amphioxus (Branchiostoma lanceolatum) NR7 nucleotide sequence with the corresponding amino acid translation. (B) Comparison of amphioxus NR7 with NR7 sequences from other species. The DNA-binding domain (DBD) (upper panel) and ligand-binding domain (LBD) (lower panel) are shown. (C) Alignment of the known sequence of Branchiostoma lanceolatum NR7 (1-389) with the sequence of Branchiostoma floridae NR7 (1-425). Sequence conservation is indicated at the bottom. Above the sequence is the LBD helix representation of the crystallographic structure and the structure predicted with AlphaFold. Fig. S2. Phylogenetic analysis of the nuclear receptor (NR) superfamily. The maximum likelihood tree corresponds to the shortened version presented Fig. 1. Classical NR subfamilies are simplified as triangles. Branch support values were assessed by approximate likelihood-ratio test (aLRT) and are plotted only if superior to 0.97, which is considered fully robust. Accession numbers are given in the Additional file 1: Data S1. Fig. S3. Developmental expression of amphioxus (Branchiostoma lanceolatum) NR7 established by whole mount in situ hybridization. Maternal expression of NR7 is detectable at the 8-cell stage (A) and remains detectable at blastula stages (B). At the gastrula stage (C), NR7 expression is in the anterior ectoderm (black arrow). Dorsal (D) and lateral (E) views of an early neurula. (F) Lateral view of a mid neurula. NR7 is expressed in the endoderm. (G) Late neurula in lateral view with NR7 expression in the cerebral vesicle of the anterior central nervous system, the gut endodern and the club-shaped gland in the pharynx. (H) Higher magnification of the region outlined in (G). Black arrow marks the signal in the cerebral vesicle and the arrowhead points to expression in the club-shaped gland. (I) Lateral view of a larva. (J-K): Higher magnification of the region outlined in (I). (J) Focus on the pharyngeal regi [file 12915_2022_1413_MOESM1_ESM.zip › 2022-09-28_BMC-Biol.supplementary-info.Beinsteiner_proof-reading.pdf]

BMC Biology

Supplementary Information for

A novel nuclear receptor subfamily enlightens the origin of heterodimerization

Additional file 1 includes: Figs. S1 to S11**,** Tables S1 to S5 and Data S1 Legend **(**Data S1 being a excel file).

Fig S2 is also provided independently as a high resolution figure

A

```
B.lanceolatum NR7 sequence

1    AACAACATGGCGAGACAGGATGACGCGCAGGCGGGCTCCACGGAGCTGGCCGCGAACAGCGAGCTGCTCGGCCTAAACCCGCCCTTCACGGACGGCCCGCCGCCGCCCTTCTGTCTCATTTGTAACGATAGGGCTTCTGGCTATCATTAC
1     N  N  M  A  R  Q  D  D  A  Q  A  G  S  T  E  L  A  A  N  S  E  L  L  G  L  N  P  P  F  T  D  G  P  P  P  P  F  C  L  I  C  N  D  R  A  S  G  Y  H  Y

151  TCGGTGTACTCTTGCGAGGGATGCAAGGGTTTCTTCAAACGGACTGTACAGAAGAACCTGTCGTACAAGTGTAAGGGGGAGGGGCATTGTGAAATCAACAAATTCACGCGAAACAACTGCCAGTCCTGTCGATTTCAGAAATGTATGGAC
51    S  V  Y  S  C  E  G  C  K  G  F  F  K  R  T  V  Q  K  N  L  S  Y  K  C  K  G  E  G  H  C  E  I  N  K  F  T  R  N  N  C  Q  S  C  R  F  Q  K  C  M  D

301  AACGGGATGAAGAAGGAAGCGGTACGAGAGGACAGGGCGCCAGGGGGGCGTCAGGGACAGAAGAGAATGCGACATTCCTACCACTACACATCCCAGGGTGATCTGGAGAGCAACTGCAATTCCACAGGCAGCACAGACAGCCCTCTTGAC
101   N  G  M  K  K  E  A  V  R  E  D  R  A  P  G  G  R  Q  G  Q  K  R  M  R  H  S  Y  H  Y  T  S  Q  G  D  L  E  S  N  C  N  S  T  G  S  T  D  S  P  L  D

451  TGCAGCTCACCCGGTGAACCAGAGAGTGATCTATTCTACTGTGATGATCTGGGGAAAACTTTAGTCAACTCCAAACCTGACCTGATCCCACGTTTGCCTGATGGACAAGCGTTTGATGTCAATGTGGCGCCGGAGCCAGGCCAGGTGAAT
151   C  S  S  P  G  E  P  E  S  D  L  F  Y  C  D  D  L  G  K  T  L  V  N  S  K  P  D  L  I  P  R  L  P  D  G  Q  A  F  D  V  N  V  A  P  E  P  G  Q  V  N

601  GTGATGATGCAGCTAGGCTACGACGAGTTGCGGATGATCATTGAGTGGGCACGAAAAGTACCTGgGTTCAACGAACTGCAAATGGAGGACAGAATGGCCCTTCTAAAGAGCTCTTTCATGGATCTGAACTGCCTCCGACTTTCTTACAGG
201   V  M  M  Q  L  G  Y  D  E  L  R  M  I  I  E  W  A  R  K  V  P  G  F  N  E  L  Q  M  E  D  R  M  A  L  L  K  S  S  F  M  D  L  N  C  L  R  L  S  Y  R

751  TGTCTGCCTTGGCTACCGAAAGTCTACTTTGGCCACGGTGTTGTGCTGTCGGTTGAGGAGACTGCGAGTCTGGGCTGGAACAGAGACATGATCACCCTGTGGGAACAGTACGTGGAGCGACTGCAGGAGATGAAGGTGGACCACGTGGAG
251   C  L  P  W  L  P  K  V  Y  F  G  H  G  V  V  L  S  V  E  E  T  A  S  L  G  W  N  R  D  M  I  T  L  W  E  Q  Y  V  E  R  L  Q  E  M  K  V  D  H  V  E

901  TTCTGTCTGCTCAATGCCTTGGTTCTGTTCTATCCAGATGCCTCTGGTTTGAAGGACAAGCCTAAGGTGACAAGTCTACAGGGTGATGTGCTGAAGAGCCTGCGCCATTACACGGTGTCCAGGTTTAACGATTCCAGAAGACATGCGAAG
301   F  C  L  L  N  A  L  V  L  F  Y  P  D  A  S  G  L  K  D  K  P  K  V  T  S  L  Q  G  D  V  L  K  S  L  R  H  Y  T  V  S  R  F  N  D  S  R  R  H  A  K

1051 ATCCTGCTGAGGCTTCCAGCGCTGCGCACCTTCAGCTCCAAGGCCCTGGAGTCGTACCTGTCCATGGCACTGGACGGGGTCCTGAAGGTGGACGAGCTGGTGACCGAGATGCTGTCCTAG
351   I  L  L  R  L  P  A  L  R  T  F  S  S  K  A  L  E  S  Y  L  S  M  A  L  D  G  V  L  K  V  D  E  L  V  T  E  M  L  S  *
```

B

NR7 DBD

```
                     1
NR7_B_lanceolatum    CLICNDRASGYHYSVYSCEGCKGFFKRTVQKNLSYKCKG----EGHCDINKFTRNNCQACRFQKCMDNGMKKEAVREDRAPGG
NR7_B_floridae       ...........................................E.........S...........................
NR7_S.purpuratus     ..V.G..GT.C....F..............Q.L.S.R.RSNM..C.E...TS..S..F.....LTA...TD......L...
NR7_H.robusta        ..V.G..G..F...TF..............SMV.A.RE....S....V..L.....Y.....LEM...........T...
NR7_C.gigas          .M.....G.......F..........S.....V.T..N......S.V..........Y...V..TQM...R.......S...
NR7_L.gigantea       ..V.E.....F....F..............A.T.V.RE....Q.N.V.......S..H..YN..VAQ..R........S...
NR7_C.teleta         ..V.G.KG..F.....T...............N.C..E....N.M.VV......S..C......IEV...R.......S...
NR7_S.kowalevskii    ..V.S.KG.......F..............Q.K.S..E....SRN.E.T.YS..S..S......VNM...ID......T...
NR7_A.californica    ....G.K........F..........S.....T.C..D....Q.M.F....S..S..F......LNM..RRD......S...
A.millepora          .VV...K.T....G.FT.............Q.E.T.......D.N.EV.QIS..R..Y...N..LAQ..L........T...
N.vectensis          .VV...K.T....G.FT.............Q.E.T.R.....NQD....QH...R..Y...E..VKS..L.Q...D..T...
```

NR7 LBD

```
                     1
NR7_B_lanceolatum    PESDLFYCDDLGKTLVNSKPDLIPRLPDGQAFDVN------------------------VAPEPGQVNVMMQLGYDELRMIIEWARKVPGFNELQMEDRMALLKSSFMDLNCLRLSYRCLPWLPK-VYFGHGVVLSVEETA
NR7_B_floridae       ......................QV....E.ST..........................................L..........................S.......................F......T..Q..
NR7_S.purpuratus     TG..VEPWRPTV.A.MDA..H...DASA.PPPGMLFSMDSMPNFMPP....SPNEFSPPPYSIRGFT.QDL..G.FQ..Y..VK..KSI...RQ.CL..Q.....A......V...A..SVDC..M.LQ.TTKCM.TIS.CK
NR7_H.robusta        STLHSPEL...I.R..DAQ.H.S.STQ.LPSYKQ-...QSL..................NSSTILEMGNL.HF..L...L..A.........GK.SPV.QI.........E..V...AF.S.DCDGC..R.TE.LEIPLQKCS
NR7_C.gigas          SKPTESFE.PIMEG.I.AR......VDGN------..................IC.....--LQSMGI.QL..Y..A..KY..D..K.....T..RL..Q..........E..V......SMETGSC.IK.AE.LKVPI.LAQ
NR7_L.gigantea       ------IY.SMIE....AQ..IF.KSESRFLSK.LFQQKRLNIRGLGYYNDKLICYYIIENFGGNIG..DL..Y..L..KF.....K.....Q.MS.D.Q.C....A..E..V...A..SMNLDRC.IK.AE...IPTDVAQ
NR7_C.teleta         .S.L.PEDE..ISQ.IEAR...T.N.DQA------....................-EENKFDI.NL..F..M...L...........PS.LI..Q.....A...E..VF..A..S.EFESG.IK.AK..I.TGD.SL
NR7_S.kowalevskii    Q.DQSKAYETMAEL.TAAR..IV.EAQSFK-----....................-DMSNFGIDDL..Y..K..QL..Q...H....RG.LI..Q.....A...E..V...A..SRSENGL..R..ANMK.TKDQAM
NR7_A.californica    DGEMAGME.A.RDQ.LAAR.H.Y.KADGQPD----....................-KM.KISI.EL..Y..L...Y..D..K.....S..E.A.Q..........E..V......SMHLEG..IK.AEDTI.PLDYCE
A.millepora          -P.SREETM-AFIAKIQENVT.V.EI.GHSLESD-....................EK.GSKD..KL.A.A.T..QFV.Q..KG....KD.HL..QVC...AC.....VF..A..SVACD.MS.R.SQNTI.EK..VV
N.vectensis          -KE.ENEFL-IYIQQLRHDV.V..DS.VKPPEGS-....................P-AAGFS.DKL..Y..Q..YQV.Q.GKN....R..KL..QIT...T......VF..A..SICCD.DSLM.AK.II.NKPQCL

NR7_B_lanceolatum    SLGWNRDMITLWEQYVERLQEMKVDHVEFCLLNALVLFYPDASGLKDKPKVTSLQGDVLKSLRHYTVSRFN-DSRRHAKILLRLPALRTFSSKALESYLSMALDGVLKVDELVTEMLS*-----
NR7_B_floridae       G.............................................................T.................................................................
NR7_S.purpuratus     D...G-.L.NATVEFCST..DLSL.VT...I..G..VCF...P..VEVDS..E..KCY.DC.QK.IA.A.PNEPK.YG...M...T..KV.A...M.QFMGLK.A.KIQ.PG..E..MNCTI...
NR7_H.robusta        DVRLGKELVK.TLEFIN..RFTNI.ST..AI.CSI..T.......A..RS.S...SRL.DC.Q...NANYPSEP..SCRL.....S...I.AH.A.MF..LS.E.NV.MND..L.IINKIG...
NR7_C.gigas          KM..GKELVAATVEFSA..K.LSI.K....I..GI..T....QN.M..Q..IL..TQI.DR..K.HSHK.PGNV..YG.M.....F...V.A..A.RF..LT...T.QLN...L..IN-.....
NR7_L.gigantea       GM..G.ELVNATIEFTS..R.L.I.RT..AI......TF...I.IQ..LQ.LG..TKI.DA..R..SRNYPTET..YG...........V.A..A.KF..LT...SIQLND..F..IT-.....
NR7_C.teleta         NI..GK.L.MFTGEF.Q..K.VQI..T.......II.T....C.IQ..R..MQ..SQIIDC.WR.CTTSYPDEP..YG.M.........V.A..A.RF...S...SI.MNA..L..M.-.....
NR7_S.kowalevskii    EI..GQEL.QSTLN..S.I..VN.......F...I..T....A..Q..TS..T..AKIVDA....IKTHYPK.AK.Y..V.....T...VA...T.RF...T...TIQIN...S..MG-.....
NR7_A.californica    NM..GKELVDGTIDFAQ..K.INL.LT...I...I..M....H...Q.LRI.EM.SRI.DC..RHI.DQQPA.NK.FG..........IV.A..ADRF..LTF..SIQLN...L..MN-.....
A.millepora          A...AE.LVDATLEFSDK.RSLNL.QN..AC.S....LS..TPE..NRA..EE..AK.NSI..D.AA.LYPLKPN.LG.L..C..T..M..E....N.VTLEFF.K.DMPP.GRSSW.KLKTAA
N.vectensis          EM..SM.LTETTLEFCAK.RNLNM.VN..SC.SG...LS...P.VV..D...E..TN.TSC..D.EMY.YPSK.N.LG.L..C..S..AY.E....N.VTLEFF.K.DMPP..A.L.E-.....
```

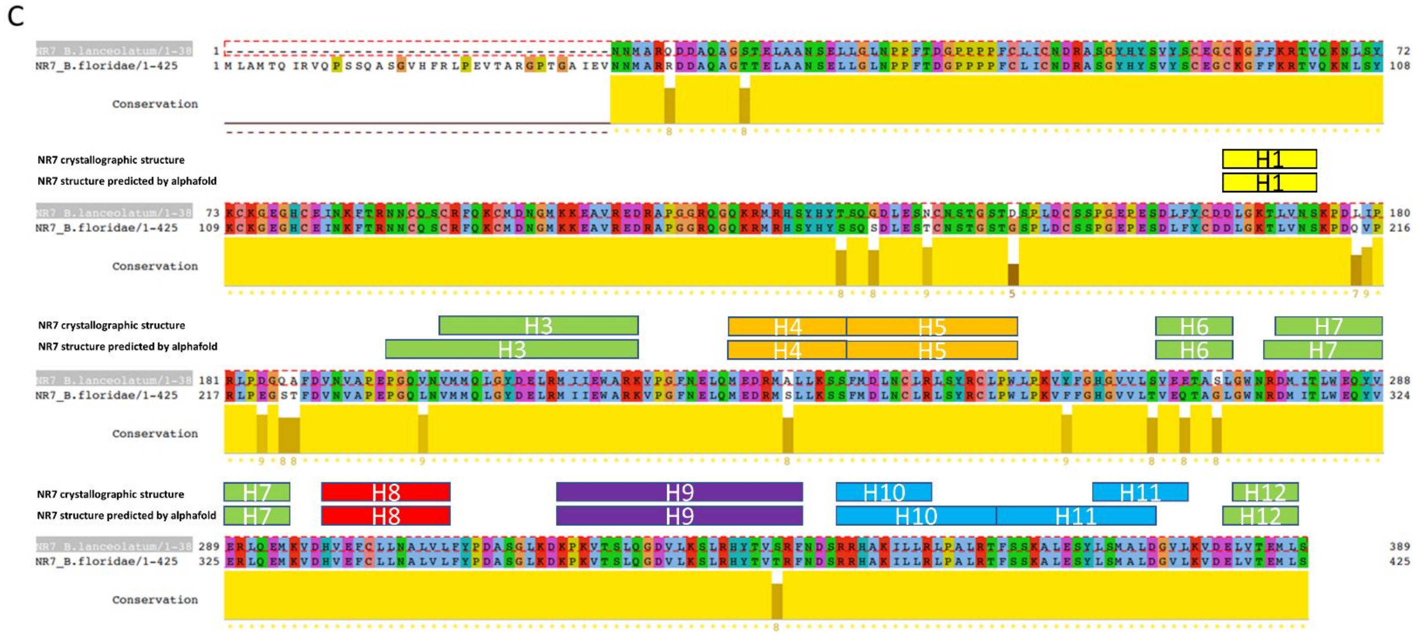

Fig. S1. Sequence of amphioxus NR7.

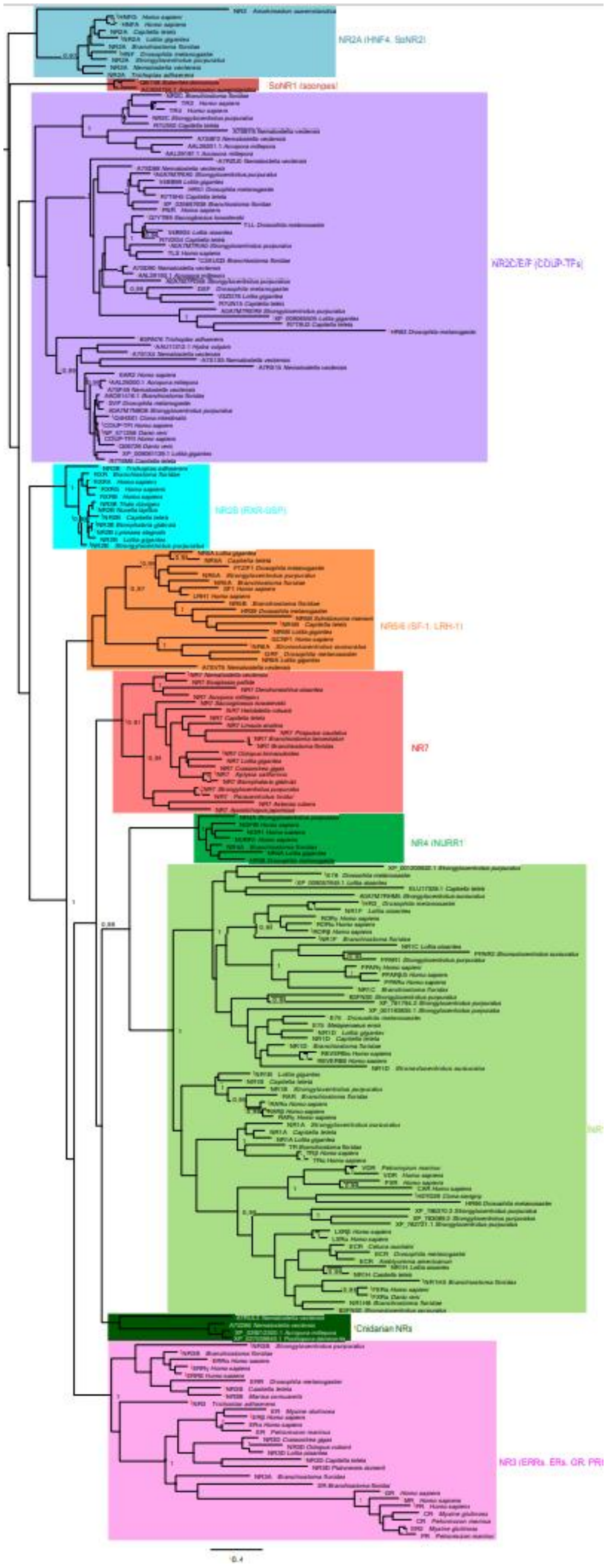

Fig. S2. Phylogenetic analysis of the nuclear receptor (NR) superfamily.

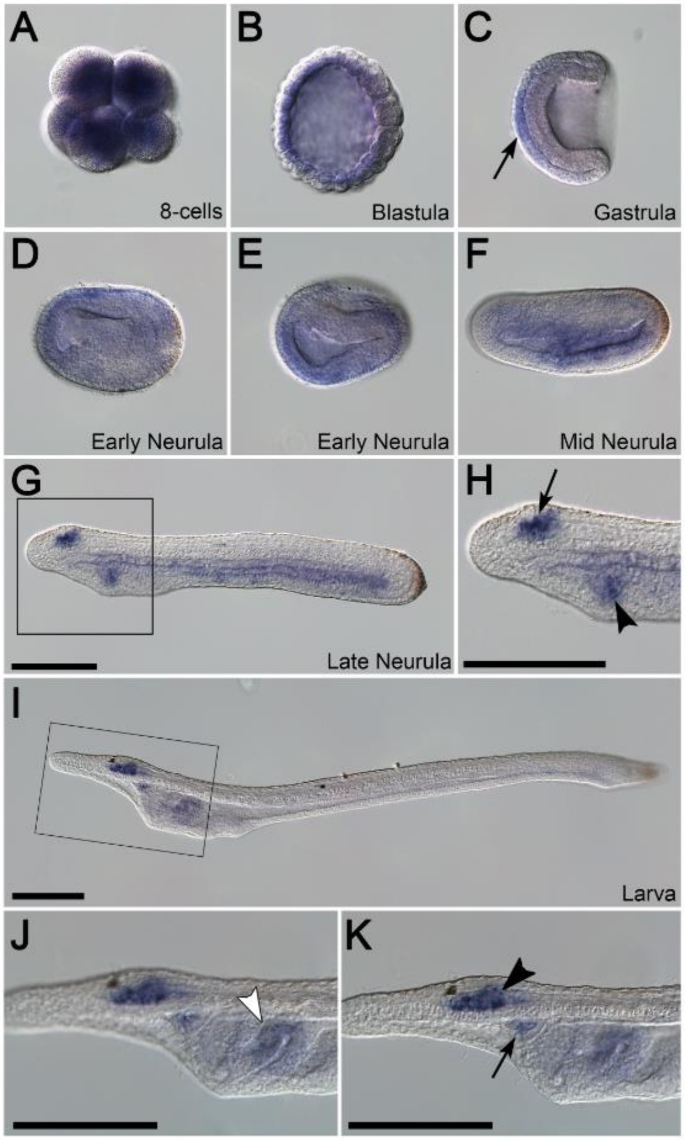

Fig. S3. Developmental expression of amphioxus (*Branchiostoma lanceolatum*) NR7 established by whole mount *in situ* hybridization.

A

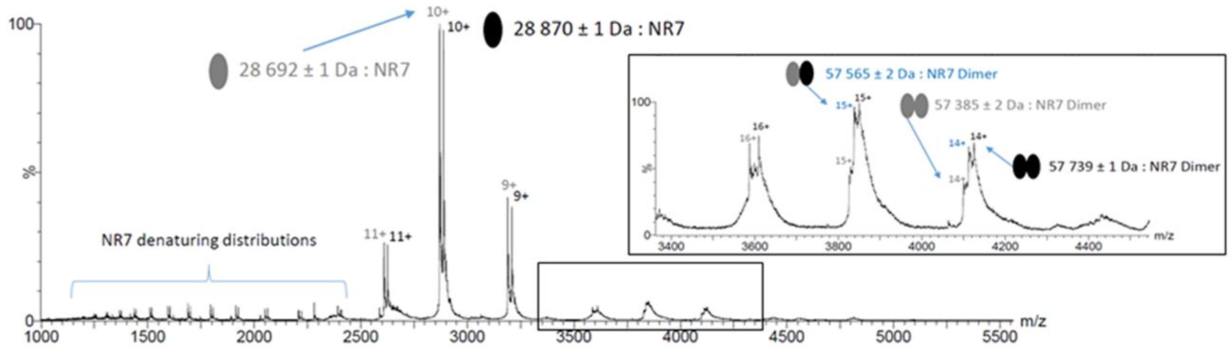

28 692 ± 1 Da : NR7
28 870 ± 1 Da : NR7
57 565 ± 2 Da : NR7 Dimer
57 385 ± 2 Da : NR7 Dimer
57 739 ± 1 Da : NR7 Dimer
NR7 denaturing distributions
100
%
0
10+
11+
9+
16+
15+
14+
m/z
1000
1500
2000
2500
3000
3500
4000
4500
5000
5500
3400
3600
3800
4000
4200
4400

B

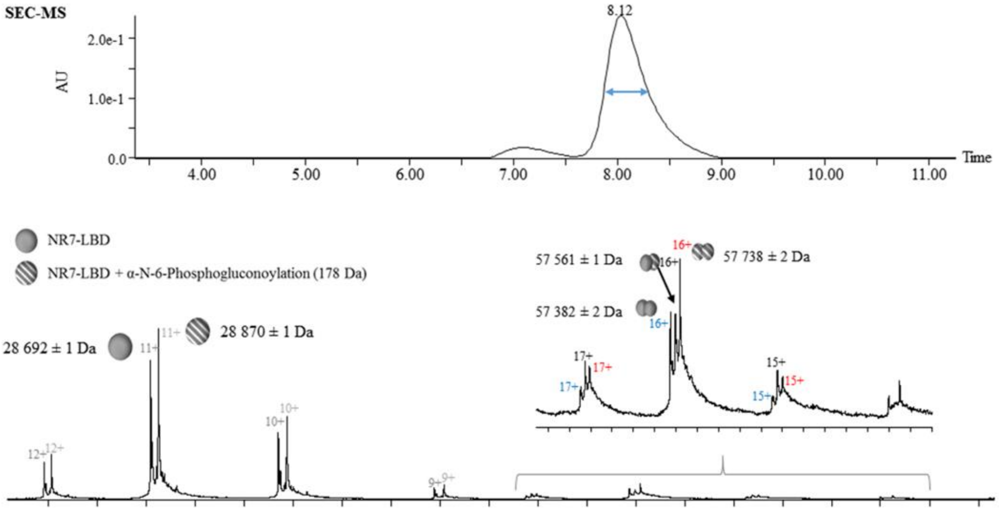

SEC-MS
8.12
AU
2.0e-1
1.0e-1
0.0
4.00
5.00
6.00
7.00
8.00
9.00
10.00
11.00
Time
NR7-LBD
NR7-LBD + α-N-6-Phosphogluconoylation (178 Da)
28 692 ± 1 Da
28 870 ± 1 Da
57 561 ± 1 Da
57 738 ± 2 Da
57 382 ± 2 Da
12+
11+
10+
9+
17+
16+
15+

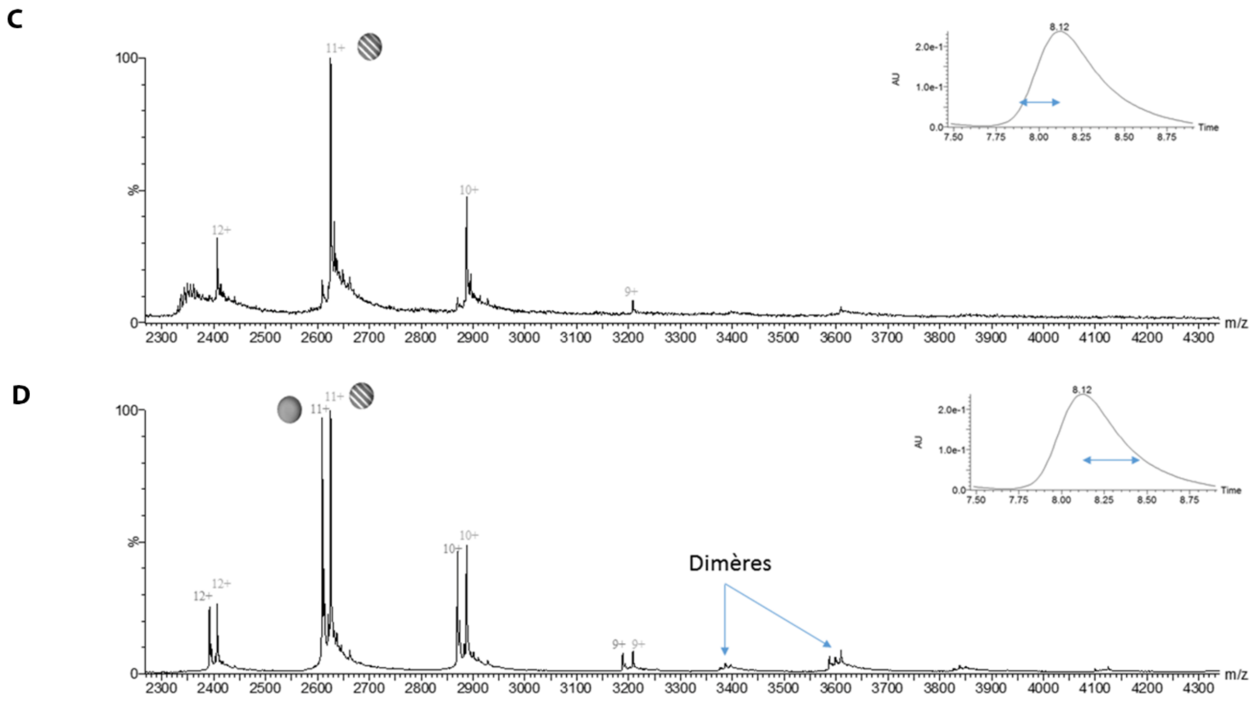

Fig. S4. Mass spectrometric analysis of amphioxus NR7.

A

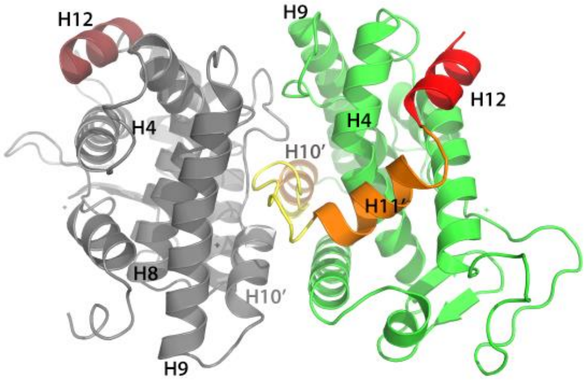

B

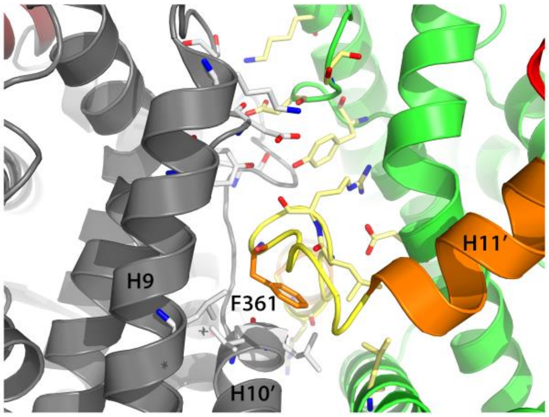

C

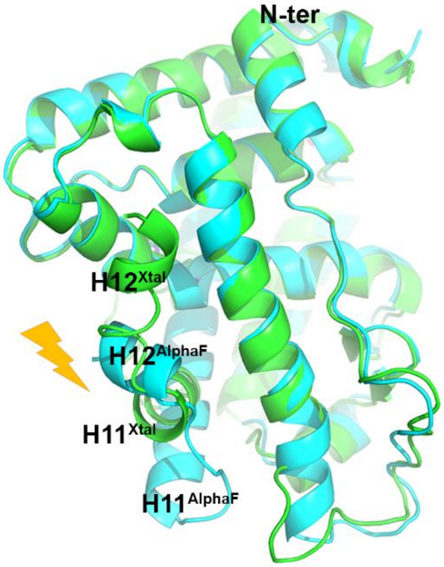

Fig. S5. **A-B**. Crystal packing effects stabilize the conformation of the loop connecting helix H10′ to H11′ in amphioxus NR7. **C.** Comparison of the crystal structure of NR7 LBD (green) with the structure predicted by AlphaFold colab (cyan).

A

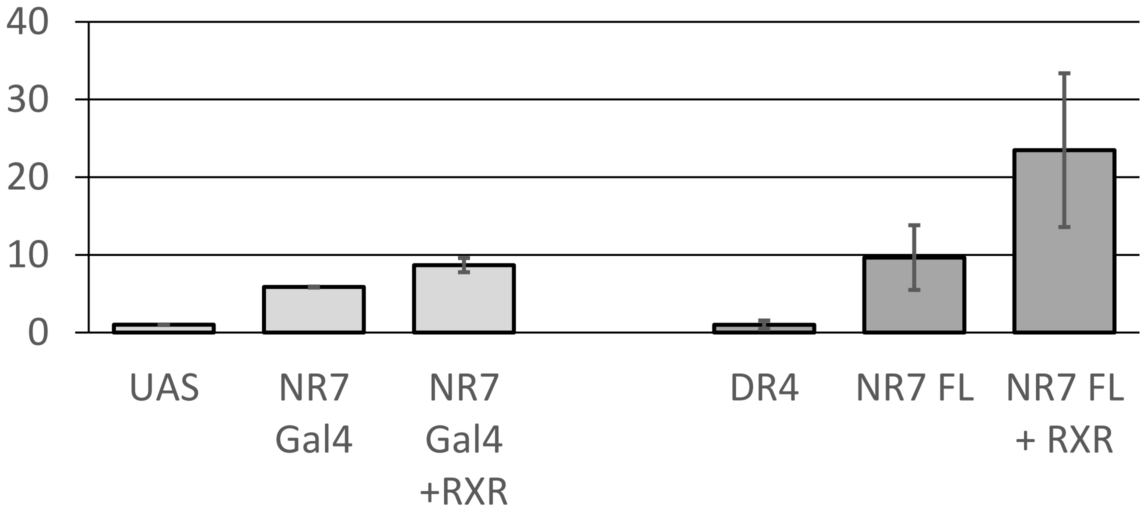

B

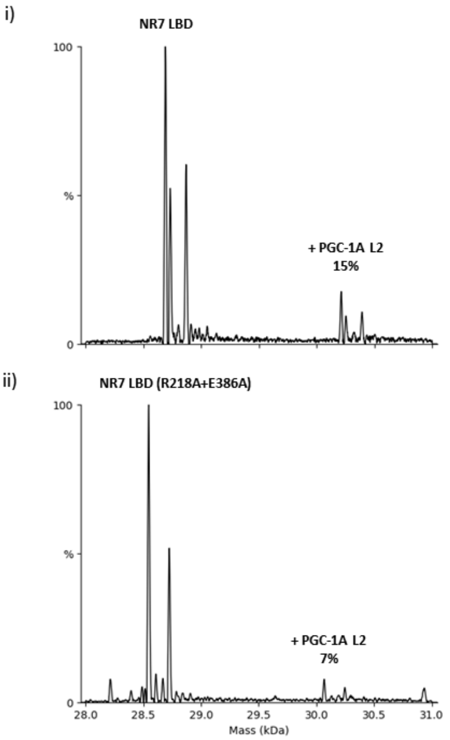

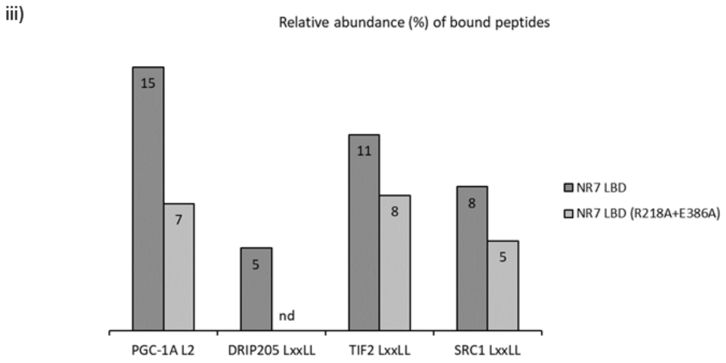

iv)

Peptide	Experimental mass (Da)	
	NR7 LBD	NR7 LBD (R218A+E386A)
-	28,691	28,548
+ PGC-1A L2 (EEPSLLKKLLLAPA)	30,213	30,068
+ DRIP205 (KNHPMLMNLLKDNPAQ)	30,553	nd
+ TIF2 (KHKILHRLLQDSS)	30,272	30,123
+ SRC1 (RHKILHRLLQEGSPS)	30,462	30,319

C

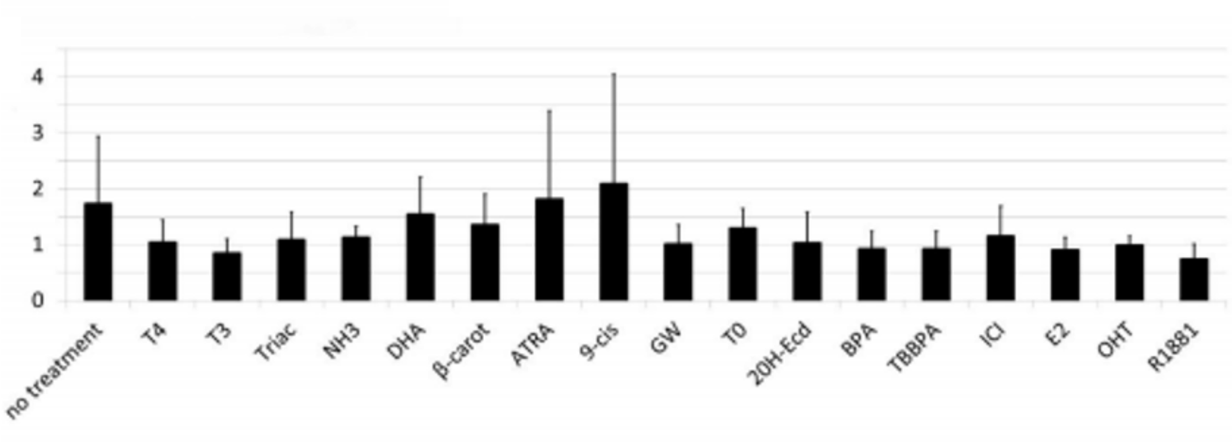

Fig. S6. Functional characterization of amphioxus NR7.

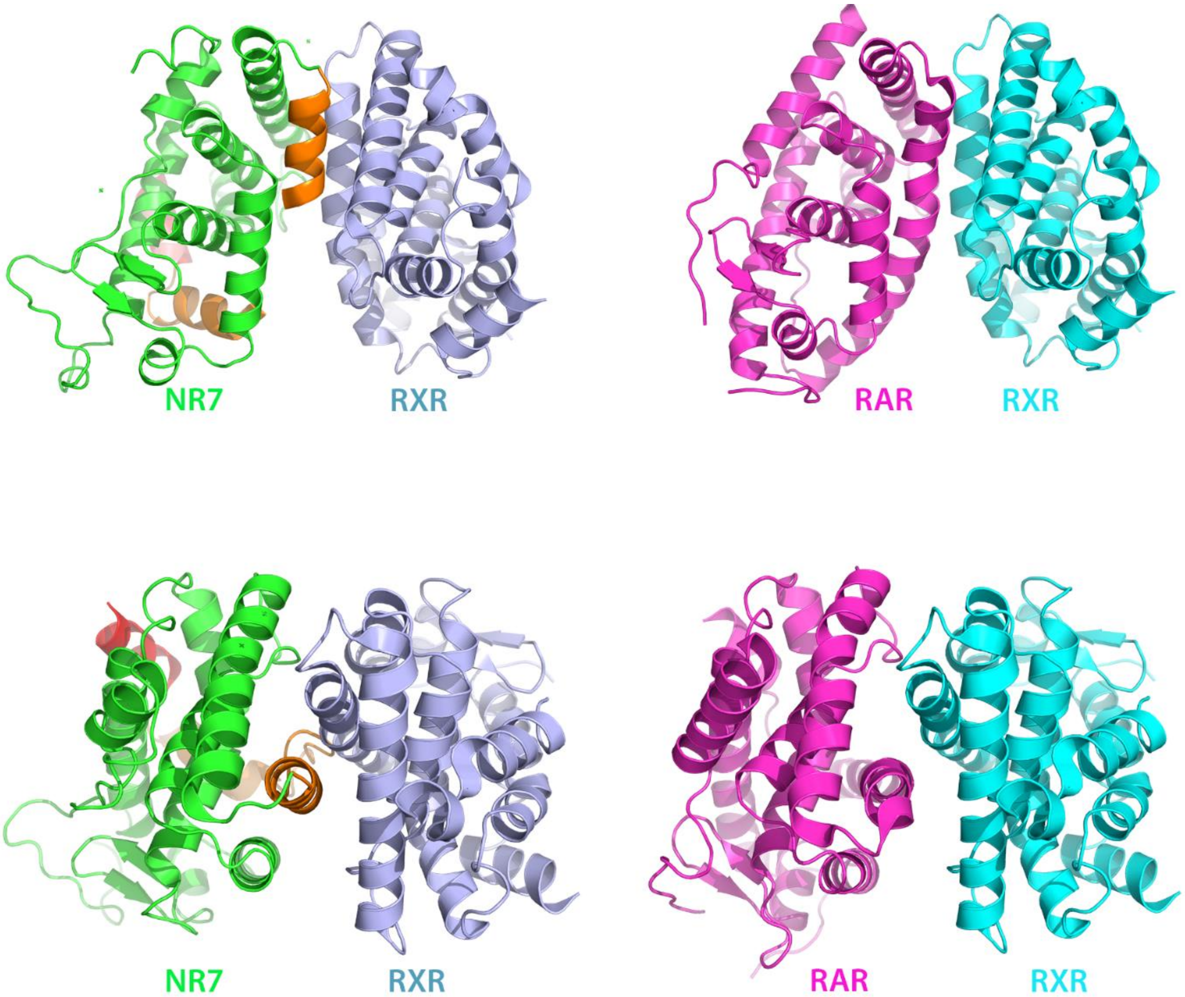

Fig. S7. Heterodimer formation of amphioxus NR7 and RXR.

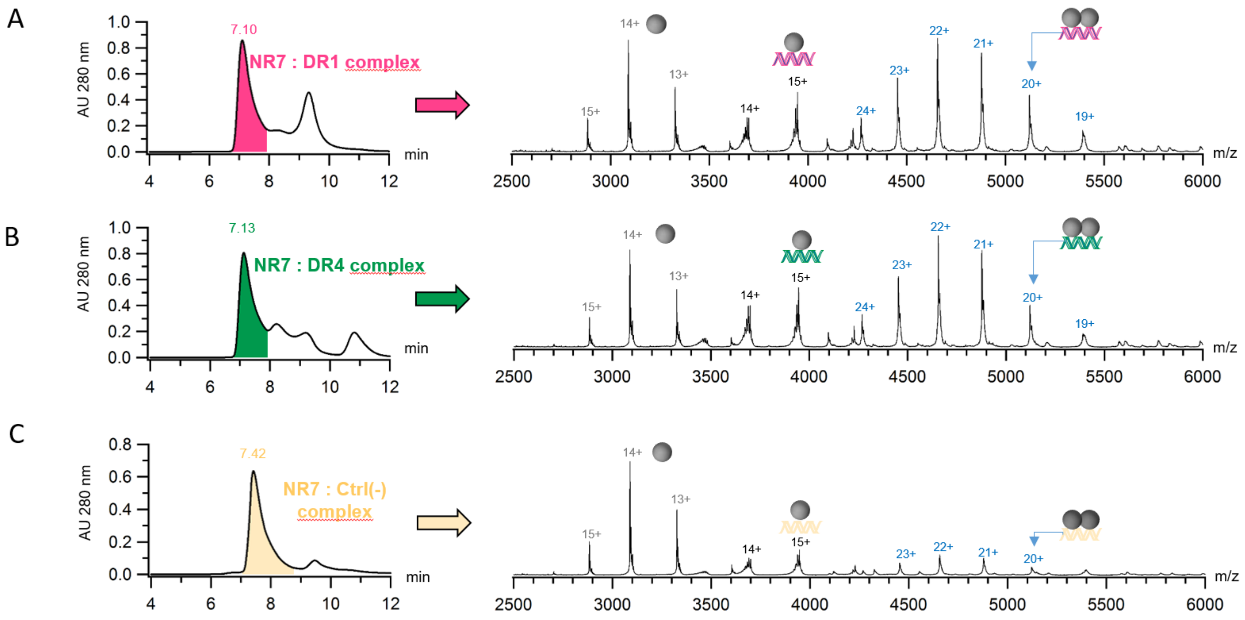

Fig. S8. Full NR7 forms a homodimer on direct repeat response elements, but not on control DNA.

.

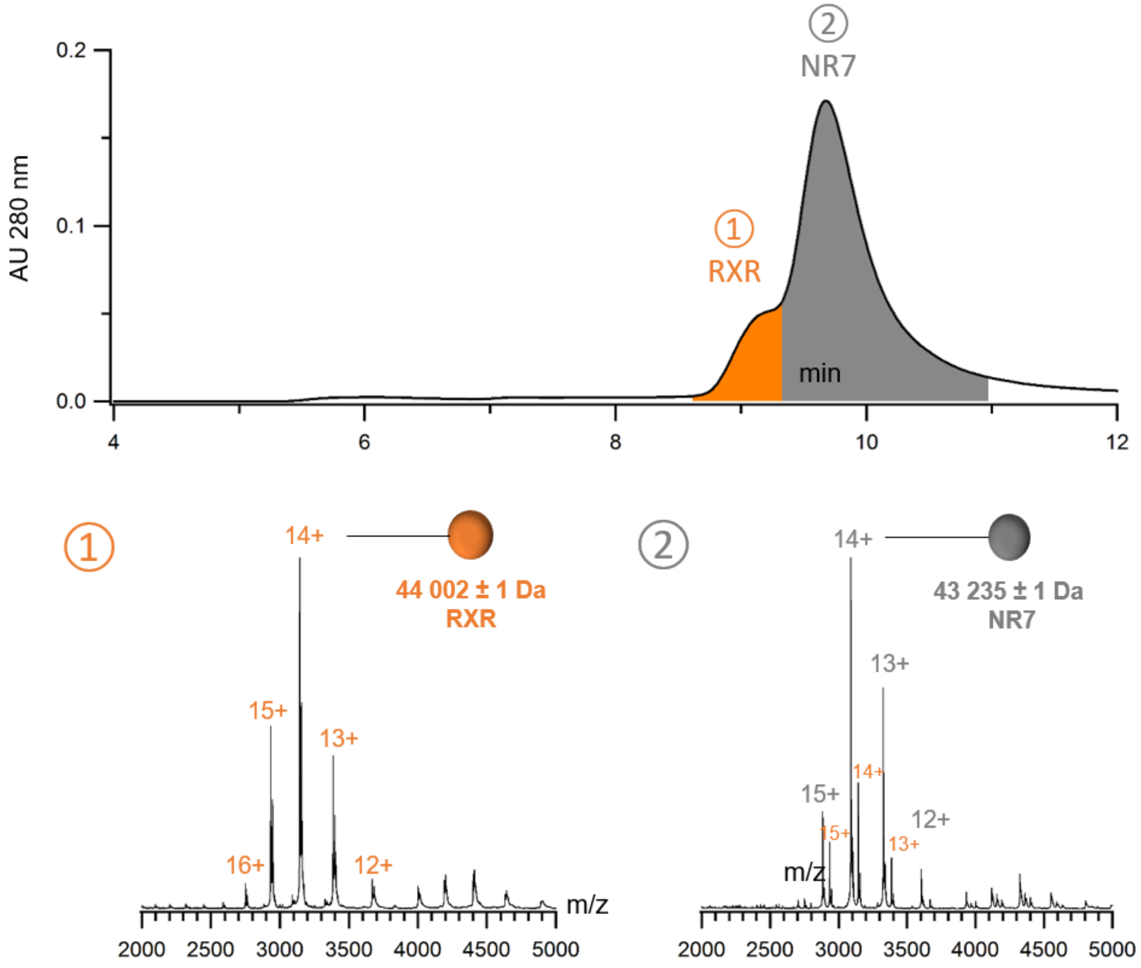

Fig. S9. Full NR7 does not heterodimerize with RXR in absence of DNA.

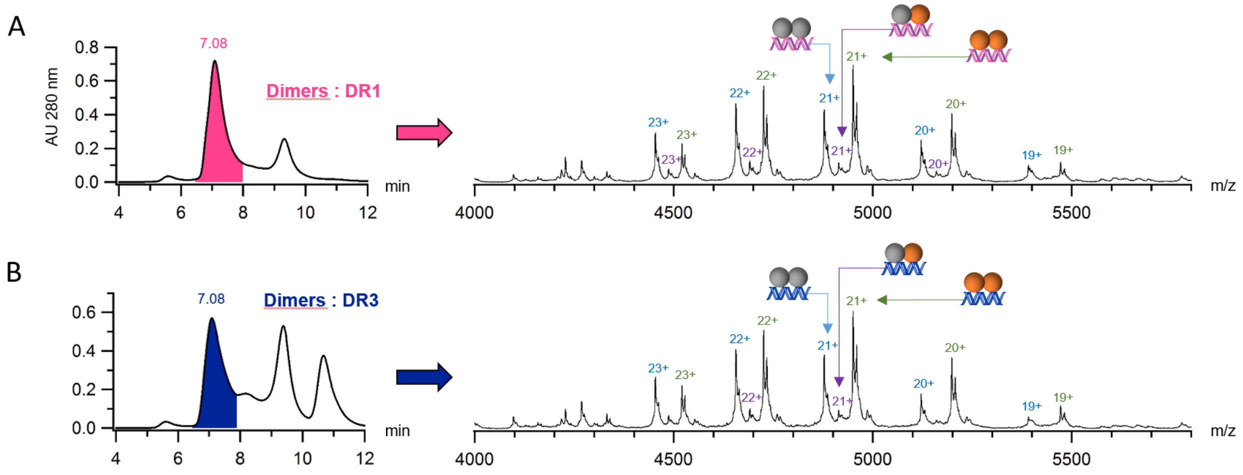

Fig. S10. Full NR7 heterodimerizes with RXR on other AGGTCA response elements organized as DR1 and DR3.

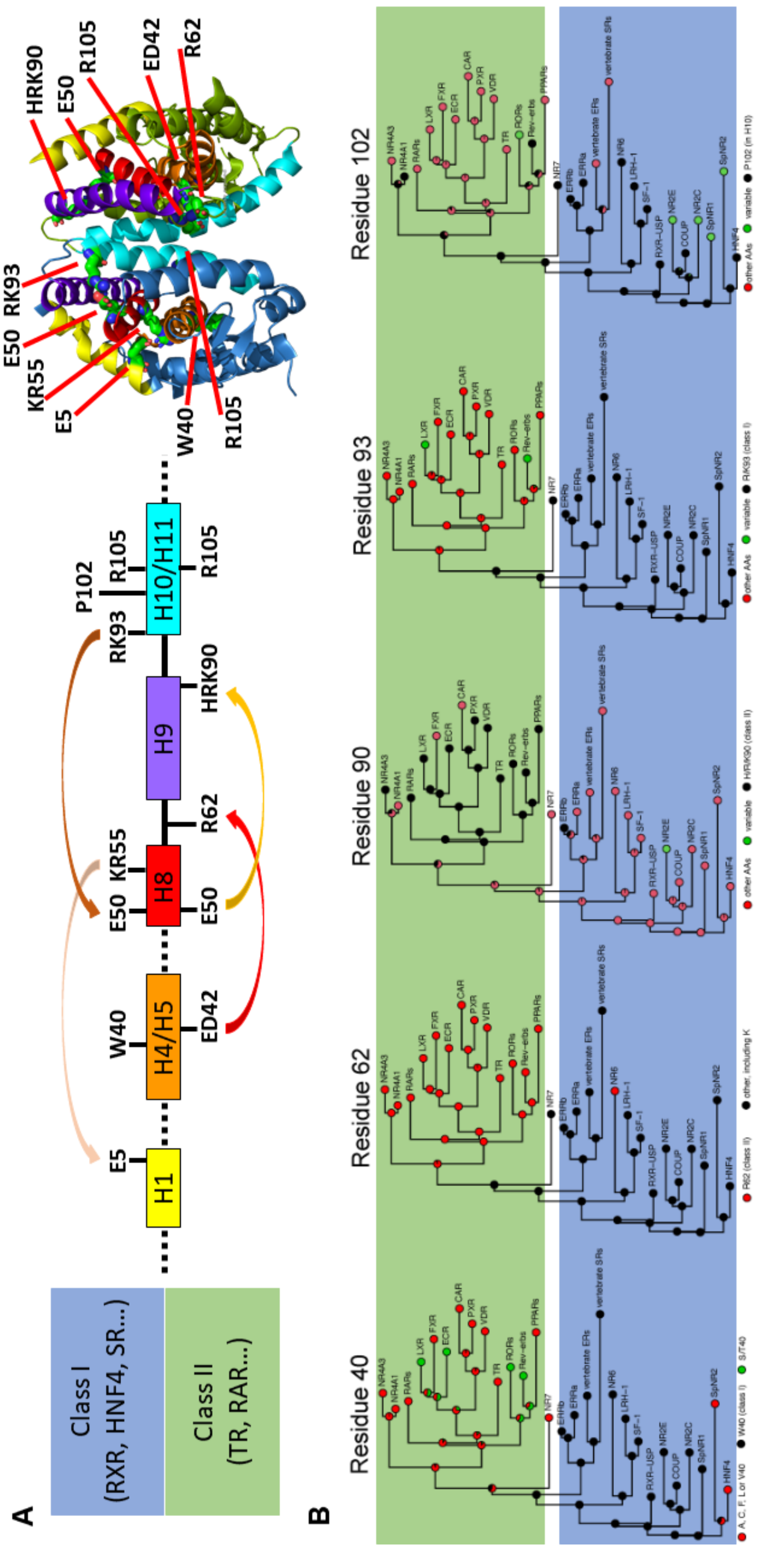

Fig. S11. Evolutionary analysis of critical class I and class II amino acids residues involved in dimerization, plus the destabilizing proline 102.

Table S1.

Theoretical masses and measured masses of the identified species relative to SEC-nMS analyses of the NR7 LBD, RXR LBD and PPAR LBD, alone or in mixture. The star relates to the observed α-N-6-phosphogluconoylation (+178 Da) modification of the N-terminal His_6-tag.

Species	Theoritical Masses (Da)	Experimental Masses (Da)
NR7-LBD	**26 638**	**28 692 ± 1**
NR7-LBD*	**26 816**	**28 870 ± 1**
NR7-LBD :NR7 dimer-LBD	**53 276**	**57 385 ± 1**
NR7-LBD :NR7-LBD* dimer	**53 454**	**57 564 ± 2**
NR7-LBD* :NR7-LBD* dimer	**53 632**	**57 740 ± 1**
RXR	**27 184**	**27 185 ± 1**
RXR*	**27 362**	**27 363 ± 1**
PPARα-LBD	**33 142**	**33 143 ± 1**
PPARα-LBD*	**33 320**	**33 320 ± 1**
RXR :PPARα-LBD	**60 326**	**60 333 ± 1**
RXR* :PPARα-LBD	**60 504**	**60 510 ± 1**
RXR :PPARα-LBD*	**60 504**	**60 510 ± 1**
RXR* :PPARα-LBD*	**60 682**	**60 687 ± 1**

** : Species + α-N-6-Phosphogluconoylation modification (178 Da)*

Table S2.

Oligonucleotides used for polyacrylamide gel electrophoresis experiments. Half-sites of the REs are underlined.

Name	Forward primer	Reverse primer
**DR0**	5’-GATTTGAGGTCAAGGTCACACAGTTA-3’	5’- TAACTGTGTGACCTTGACCTCAAATC-3’
**DR1**	5’-GATTTGAGGTCAGAGGTCACACAGTT-3’	5’- AACTGTGTGACCTCTGACCTCAAATC-3’
**DR3**	5’-GATTTGAGGTCACAGAGGTCACACAG-3’	5’- CTGTGTGACCTCTGTGACCTCAAATC-3’
**DR4**	5’-ATTTGAGGTCACAGGAGGTCACACAG-3’	5’- CTGTGTGACCTCCTGTGACCTCAAAT-3’
**IR3**	5’-GATTTGAGGTCACAGTGACCTCACAG-3’	5’- CTGTGAGGTCACTGTGACCTCAAATC-3’
**Ctrl(-)**	5’-TGGCCCGACACTCACTGTTTGGATGA-3’	5’-TCATCCAAACAGTGAGTGTCGGGCCA -3’

Table S3.

Measured masses of the identified species relative to SEC-nMS analyses of full NR7 with DR1, DR4 and Ctrl(-) response elements **(Fig. S8).** Masses corresponding to full NR7 alone or bound to DNA as monomer or homodimer are reported in this table.

Analysis	NR7	NR7:DNA	$(NR7)_2$:DNA
**NR7 + DR1**	43 233 ± 1 Da	59 178 ± 1 Da	102 415 ± 2 Da
**NR7 + DR4**	43 234 ± 1 Da	59 178 ± 1 Da	102 413 ± 1 Da
**NR7 + Ctrl(-)**	43 234 ± 1 Da	59 178 ± 1 Da	102 414 ± 1 Da

Table S4.

Measured masses of the identified species relative to SEC-nMS analyses of full NR7 with DR0, DR4, IR3 and Ctrl(-) response elements (**Fig. 5**). Masses corresponding to full NR7, RXR alone, NR7 homodimers, NR7-RXR heterodimer and RXR homodimers all bound to DNA are reported in this table (n.d. : not detected).

Analysis	NR7	RXR	$(NR7)_2$:DNA	NR7:RXR:DNA	$(RXR)_2$:DNA
**NR7 + RXR + DR0**	-	-	102 409 ± 1 Da	103 176 ± 1 Da	103 944 ± 1 Da
**NR7 + RXR + DR4**	-	-	102 412 ± 1 Da	103 179 ± 1 Da	103 946 ± 1 Da
**NR7 + RXR + IR3**	-	-	102 409 ± 1 Da	-	103 943 ± 1 Da
**NR7 + RXR + Ctrl(-)**	43 234 ± 1 Da	44 000 ± 1 Da	-	-	103 947 ± 2 Da

Table S5.

Measured masses of the identified species relative to SEC-nMS analyses of full NR7 with DR1 and DR3 response elements (as shown in **Fig. S10**).

Analysis	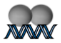 $(NR7)_2$:DNA	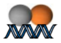 NR7:RXR:DNA	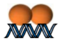 $(RXR)_2$:DNA
**NR7 + RXR + DR1**	102 412 ± 1 Da	103 178 ± 2 Da	103 946 ± 1 Da
**NR7 + RXR + DR3**	102 413 ± 1 Da	103 178 ± 2 Da	103 946 ± 1 Da

Data S1.

Accession number for sequences used in the tree of Fig. S1. Accession numbers are coming mainly from GenBank, and alternatively from UniProt or from the website of the Joint Institute of Genomics (JGI). For the four species where sequences are coming from the JGI, they can be accessed using the following species-specific search pages:
https://genome.jgi.doe.gov/pages/search-for-genes.jsf?organism=Capca1
https://genome.jgi.doe.gov/pages/search-for-genes.jsf?organism=Helro1
https://genome.jgi.doe.gov/pages/search-for-genes.jsf?organism=Lotgi1
https://genome.jgi.doe.gov/pages/search-for-genes.jsf?organism=Nemve1
