# Supplementary figures and images for: A novel nuclear receptor subfamily enlightens the origin of heterodimerization
Source: BMC Biol. 2022 Oct 5;20:217. doi: 10.1186/s12915-022-01413-0 (PMC9535869; doi:10.1186/s12915-022-01413-0)

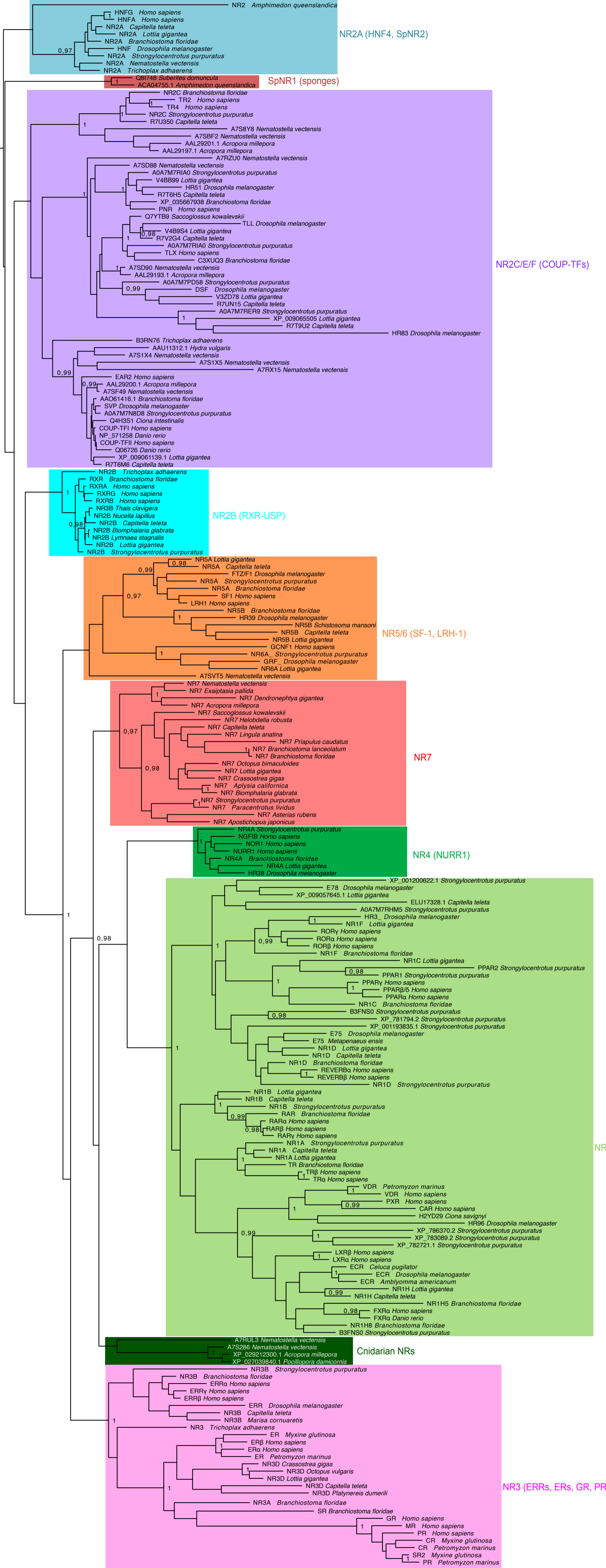

Supplement: Supplementary file 1 — Additional file 1: Fig. S1. Sequence of amphioxus NR7. (A) Amphioxus (Branchiostoma lanceolatum) NR7 nucleotide sequence with the corresponding amino acid translation. (B) Comparison of amphioxus NR7 with NR7 sequences from other species. The DNA-binding domain (DBD) (upper panel) and ligand-binding domain (LBD) (lower panel) are shown. (C) Alignment of the known sequence of Branchiostoma lanceolatum NR7 (1-389) with the sequence of Branchiostoma floridae NR7 (1-425). Sequence conservation is indicated at the bottom. Above the sequence is the LBD helix representation of the crystallographic structure and the structure predicted with AlphaFold. Fig. S2. Phylogenetic analysis of the nuclear receptor (NR) superfamily. The maximum likelihood tree corresponds to the shortened version presented Fig. 1. Classical NR subfamilies are simplified as triangles. Branch support values were assessed by approximate likelihood-ratio test (aLRT) and are plotted only if superior to 0.97, which is considered fully robust. Accession numbers are given in the Additional file 1: Data S1. Fig. S3. Developmental expression of amphioxus (Branchiostoma lanceolatum) NR7 established by whole mount in situ hybridization. Maternal expression of NR7 is detectable at the 8-cell stage (A) and remains detectable at blastula stages (B). At the gastrula stage (C), NR7 expression is in the anterior ectoderm (black arrow). Dorsal (D) and lateral (E) views of an early neurula. (F) Lateral view of a mid neurula. NR7 is expressed in the endoderm. (G) Late neurula in lateral view with NR7 expression in the cerebral vesicle of the anterior central nervous system, the gut endodern and the club-shaped gland in the pharynx. (H) Higher magnification of the region outlined in (G). Black arrow marks the signal in the cerebral vesicle and the arrowhead points to expression in the club-shaped gland. (I) Lateral view of a larva. (J-K): Higher magnification of the region outlined in (I). (J) Focus on the pharyngeal regi [file 12915_2022_1413_MOESM1_ESM.zip › FigS2_V2.pdf]
